# Revealing unexplored bacterial and fungal variability in interconnected Antarctic brines

**DOI:** 10.1016/j.crmicr.2025.100538

**Published:** 2025-12-18

**Authors:** Maria Papale, Ciro Sannino, Dario Battistel, Gianmarco Mugnai, Luigimaria Borruso, Angelina Lo Giudice, Benedetta Turchetti, Maurizio Azzaro, Pietro Buzzini, Mauro Guglielmin

**Affiliations:** a Institute of Polar Sciences, National Research Council, Spianata S. Raineri. 86, 98122, Messina, Italy; b Department of Agricultural, Food and Environmental Sciences, University of Perugia, Borgo XX Giugno 74, 06121 Perugia, Italy; c Department of Environmental Sciences, Informatics and Statistics, University Ca’ Foscari of Venice, Via Torino, 155, 30172, Mestre, VE, Italy; d Department of Agronomy Food Natural Resources Animals and Environment, University of Padova, Viale dell’Università 16, 35020, Legnaro, Padova, Italy; e Faculty of Science and Technology, Free University of Bozen-Bolzano, Piazza Università 5, 9100, Bozen-Bolzano, Italy; f Department of Theoretical and Applied Sciences, Insubria University, Via Dunant, 3, 21100 Varese, Italy

**Keywords:** Antarctica, Brines, Fungal and Bacterial variability, Geochemical gradients, Ecosystems

## Abstract

Subsurface Antarctic brines represent highly selective habitats shaped by extreme physicochemical conditions. Here, the microbial communities of three hypersaline brines (B2, BCR, BCM) from Boulder Clay, Northern Victoria Land, which differ in salinity, pH, trace elements, and glaciological context, have been characterized. High-throughput sequencing was used to reveal that each brine hosts distinct bacterial and fungal assemblies, shaped by local environmental filters. Despite their proximity and some connectivity, microbial communities showed strong taxonomic differentiation and habitat-associated structuring, with significant divergence in diversity and indicator taxa. B2, the most saline, hosted halotolerant bacteria and many unclassified fungi, indicating strong selection and the presence of under-sampled lineages. BCM, enriched by lithogenic input, was dominated by cold-adapted yeasts, while BCR, affected by glacial meltwater, had higher microbial diversity and was dominated by terrestrial taxa.

This is the first study integrating microbial, geochemical, and geomorphological data across interconnected Antarctic brines, highlighting how fine-scale environmental heterogeneity and filtered connectivity shape microbial differentiation.

## Highlights

• Interconnected Antarctic brines show distinct bacterial and fungal communities.
• Fine-scale geochemistry and substrate context filter microbial assembly.
• B2 brine (hypersaline) shows halotolerant bacteria and many unclassified fungi.
• BCM brine shows permafrost/mineral imprint dominated by Patescibacteria and *Mrakia*.
• BCR brine shows the highest diversity of soil- and glacier-derived microbial taxa.

## Graphical abstract

**Figure:**
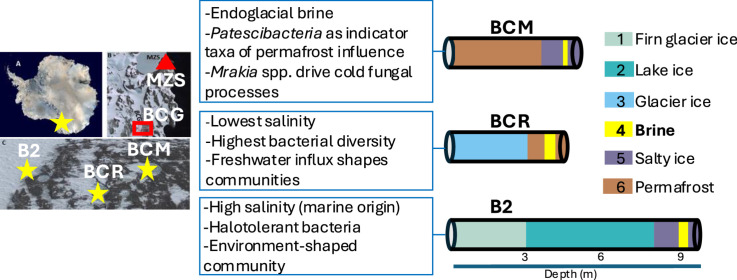

## 1 Introduction

**Fig. 1:**
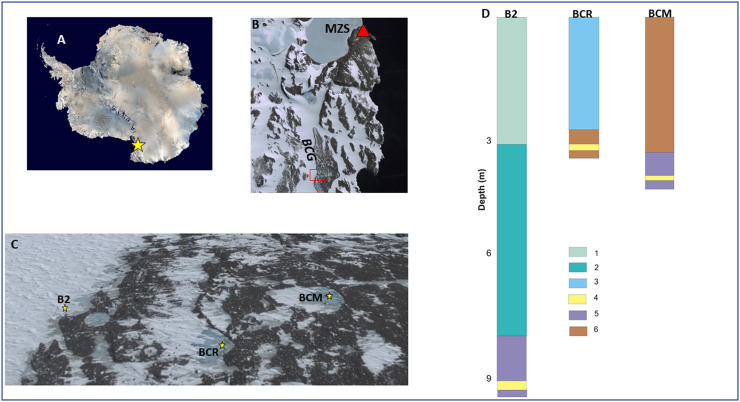
Location of the study area and main characteristics of the brine sites. A: Antarctic map (modified from https://www.coolantarctica.com/gallery/scenic/views_of_antarctica.php) with the location of the Northern Victoria Land (orange star); B: ortophoto (02/12/2004) taken by https://earth.google.com/web/search (research 15/04/2025) of the Mario Zucchelli station (red triangle) and the Boulder Clay Glacier (BCG) with the nearby till covered area. The small red rectangle indicates the study area of C) where the three yellow stars locate the three boreholes from west to east: B2, BCR and BCM where the saline brines were found and sampled. D: main characteristics of the boreholes B2, BCR and BCM: depth and thickness of the main glaciological units. Legend: 1) firn and very recent glacier ice (post-medieval, see Forte et al., 2024); 2) lake ice; 3) recent glacier ice, (pre-medieval), 4) brines; 5) salty ice (RGP, see Forte et al., 2024); 6) permafrost (frozen till).

Antarctic briny systems, generally encapsulated within lakes (Murray et al., 2012; Papale et al., 2021) and, subordinately glaciers (Guglielmin et al., 2023; Mikucki et al., 2009; Mikucki and Priscu, 2007), represent unique environments that show a harsh combination of conditions, especially the association of high salinity and low temperatures, which makes these ecosystems particularly selective for all forms of life, including microbial ones (Guglielmin et al., 2023; Papale et al., 2021). In recent years, the Boulder Clay (BC) area in Northern Victoria Land, Antarctica, has gained significant attention due to the discovery of multiple subsurface brine pockets beneath its glacier and surrounding permafrost. These brines, labeled as B2, BCR, and BCM (Fig. 1), are believed to have originated from the cryo-concentration of marine-derived ice from the Ross Glacial Platform (Forte et al., 2024). Geophysical surveys, including Ground Penetrating Radar (GPR) and electromagnetic methods, have revealed the presence of these brines at shallow depths, indicating active hydrological systems within glaciers and permafrost (Forte et al., 2024). Their chemical composition is variable: B2 and BCM exhibit signs of intense cryo-concentration, while BCR appears to be diluted by glacial meltwater, suggesting different degrees of interaction with the surrounding environment (Forte et al., 2024). Such variations in chemical profiles could imply distinct flow patterns and degrees of interconnectivity among the brine systems.

Microbial life in these extreme environments has been a subject of extensive research. Culture-independent methods, including next-generation sequencing (NGS), have been applied to examine both the bacterial and fungal (including yeast life forms) diversity, abundance, composition, assessing the significant influence of environmental parameters (e.g., salinity and metal concentrations) on the microbial community structure and activities (Antunes et al., 2020; Borruso et al., 2018; Guglielmin et al., 2023; Lo Giudice et al., 2021; Martin and McMinn, 2018; Papale et al., 2021; Rizzo et al., 2020; Sannino et al., 2020). Notably, those studies have shown that even geographically proximate brine systems can harbor distinct microbial communities due to differences in their physicochemical stressors. For instance, variations in salinity, nutrient availability, and trace metal concentrations can lead to the selection of specific microbial taxa adapted to those conditions (Guglielmin et al., 2023; Sannino et al., 2020). Geographically proximate brine systems may host distinct microbial communities due to localized physicochemical stressors. At the same time, the existence of interconnected subsurface networks can affect microbial dispersal and environmental adaptation, potentially facilitating the exchange of microbial taxa or genetic material across different niches (Azzaro et al., 2021).

Although significant insights have been gained, critical uncertainties still persist regarding geographically proximate brine systems, characterized by variable morphology, depth, substrate composition, and glaciological context, which also exhibit corresponding differences in their microbial community structure. Recent geochemical evidence suggests that some of them (e.g., B2 and BCR) may be hydrologically connected, while BCM remains isolated due to different flow paths and substrate interactions (Forte et al., 2024). These contrasting flow patterns and geological contexts may act as environmental filters, driving microbial divergence even across small spatial scales.

This research examines the microbial community composition of three hypersaline brines (B2, BCM, and BCR), which, despite having some chemical similarities, differ significantly in their geomorphological and glaciological settings (Forte et al., 2024; Guglielmin et al., 2023). By combining microbial profiling with geochemical and geological data, the study aims to explore the origins, connectivity, and dynamics of these brines. A key goal is to determine whether microbial diversity is mainly affected by physical isolation or by shared environmental pressures. Comparing the chemical and biological signatures of the brines will reveal whether they harbour unique or shared microbial communities, offering insights into the processes that affect bacterial and fungal diversity in extreme environments.

## 2 Materials and methods

### 2.1 Study sites and sample collection

The three sites where brines B2, BCR, and BCM were found and sampled are relatively close to each other (<300 m) and are located on the Boulder Clay Glacier (BCG) and on nearby till coverage. BCG is a coastal cold-based glacier roughly parallel to the coast and ca 6 km southward of the Italian Antarctic station Mario Zucchelli (MZS). Although the three sites lie close together on the surface, they differ significantly in morphology and glaciological conditions: B2 is located on the border of a coastal glacier, BCR is situated on a perennially frozen lake, and BCM is located in a frost mound within another perennially frozen lake. The brines were found at different depths (Fig. 1) and, importantly, within different substrates. B2 is the deepest (9.1 m), followed by BCM (3.94 m) and BCR (3.2 m). Both B2 and BCM brines flow within the salty ice of the Ross Glacial Platform (RGP), whereas in BCR, brines flow within frozen till. Considering the elevations of the brines from west to east, they are at 142.8 m (B2), 146.9 m (BCR), and 145.4 m (BCM).

These three sites were studied through geophysical investigations that proved that the brines are interconnected (Forte et al., 2024). The same study underlined also that the nature of the underlying structure is different because while B2 was cored on the BCG down to the frozen till overlying on the remnants of the RGP, the BCR was cored on lake ice overlying the frozen till above the contact between BCG and RGP and finally the BCM was cored through a frost mound occurring on the frozen till directly over the RGP (Fig. 1). All the boreholes were cored with a drilling machine using a 101 mm diameter simple core auger with Widia/Diamond bites and cold compressed conditioned air as refrigerant fluid. All the cores were drilled between the middle of November and early December 2019, in particular BCR on 23/11/2019, B2 on 26/11/2019, and BCM on 1/12/2019. pH and salinity were measured with a multiparametric probe (Hanna Instruments - HI98194 model) directly in the boreholes, while gas abundance was only visually estimated.

Brine samples were collected at different depths by a peristaltic pump and sterilized tubing. During the coring in each borehole immediately before (1–2 cm above) the brine pocket, the drilling was stopped, and after that, the peristaltic syringe pump was inserted, with which three syringe tubes were filled, constituting three subsamples for each brine. Three sub-samples per brine were stored in sterile 50 ml polyethylene tubes and kept at 4 °C in the dark until transfer and laboratory processing in Italy. These three subsamples were mixed in the laboratory, and from this mixture, 5 replicates were sampled for each brine for further analyses.

### 2.2 Chemical analyses

The brines were filtered using a PTFE membrane with a pore size of 0.45 μm and diluted in ultra-pure water to ensure they fell within the calibration range in the laboratory of Ca’ Foscari University (Venice, Italy). Ion chromatography (Metrohm 761 Compact IC Chromatography) was used for analysing anions and cations. Trace and rare elements analysis was performed by ICP-MS using an iCAP RQ instrument (Thermo Scientific). Total carbon and nitrogen were determined using a Flash 2000 HT Elemental Analyzer (Thermo Scientific). Further details are published in Forte et al. (2024).

### 2.3 DNA extraction and sequencing

Five replicates for each brine were carefully filtered to collect microbial biomass onto a sterile cellulose acetate filter with a pore size cut-off of 0.2 µm (Sartorius Stedim, Biotech, Gottingen, Germany). A total of 15 filters were used (5 per brine). The DNA was extracted from these filters using the Power Water DNA Isolation Kit (Qiagen, Hilden, Germany). DNA concentrations were quantified with a Qubit 3.0 Fluorometer Assay (Life Technologies Corporation, Carlsbad, CA, USA).

Amplicons of the bacterial 16S V3 and V4 region were generated using the primers IlluAdp_16S_341f 5′-CCTACGGGNGGCWGCAG-3′ and IlluAdp_16S_805r 5′-GGACTACHVGGGTATCTAATCC-3′ (Klindworth et al., 2013). Amplicons of the fungal internal transcribed spacer region 2 (ITS2) were generated using the primers IlluAdp_ITS3_NeXTf5′-CATCGATGAAGAACGCAG-3′ and IlluAdp_ITS4_NeXTr5′-TCCTCCGCTTATTGATATGC-3′ (Tedersoo et al., 2015). The PCR products were sequenced on the Illumina MiSeq platforms, following the standard protocols of IGA Technologies Services (Udine, Italy).

### 2.4 Bioinformatics and statistical analyses

The quality of raw data was assessed with FastQC (Andrews, 2010). The sequence data underwent pre-processing, quality filtering, trimming, denoising, merging, modeling, and analysis using DADA2 in the QIIME2 environment (Bolyen et al., 2019). Chimeras were eliminated through the ‘consensus’ approach (Callahan et al., 2016). Sequence variants were clustered into operational taxonomic units (OTUs) using VSEARCH with a 97 % cutoff (Rognes et al., 2016). SILVA (version 138) and UNITE+INSD (version 9.0) were used as the reference databases for the bacterial and fungal taxonomy annotations against the representative sequences (Glöckner et al., 2017; Nilsson et al., 2019; Quast et al., 2012). Sequences were archived in the NCBI SRA database linked to BioProject accession number PRJNA1297434.

Statistical analyses were performed by the open-source software R version 4.03 (R core Team, 2024). Graphical representations were generated with the R package “ggplot2” (Wickham, 2016). Differences in microbial alpha diversity (Richness and Shannon-H indices) were assessed using ANOVA, followed by Tukey’s post-hoc pairwise multiple comparison procedure. Microbial beta diversity was calculated using the permutational multivariate analysis of variance (PERMANOVA) by the Adonis function in the R package “vegan”(Oksanen et al., 2015), while NMDS ordination, based on the Bray-Curtis distance, to visualize brine samples was performed on Past software version 4.09. Before to generate the NMDS the data were square root transformed.

To determine whether differences in community composition across brines were driven by deterministic (environmental selection) or stochastic (dispersal) processes, a null model approach based on the Raup–Crick metric was applied. OTU tables for both bacteria and fungi were first converted to presence/absence data and pooled at the brine level, considering an OTU as present only if it appeared in at least one biological replicate. Pairwise community similarity among brines was then calculated using Jaccard similarity from these presence/absence matrices. To assess whether the observed differences deviated from random expectations, an incidence-based Raup–Crick (RC) null model was applied to the same matrices, using the raupcrick function in the R vegan package with 999 randomizations. This null model maintains sample richness and OTU occurrence rates while randomly assigning OTU identities, thereby allowing evaluation of whether community dissimilarities are larger or smaller than expected by chance.

**Fig. 2:**
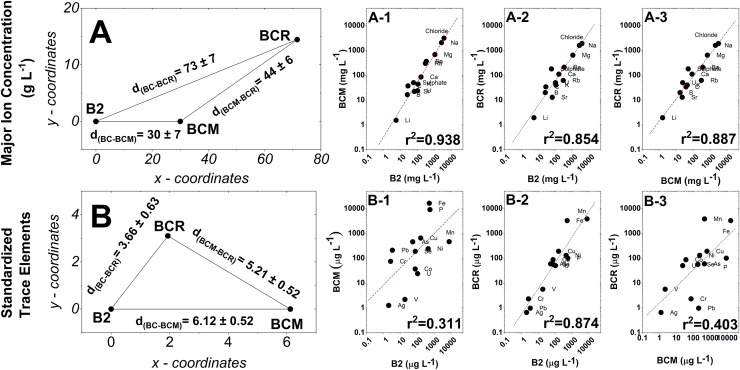
Multivariate Euclidean distance between the three brines calculated using concentration values (A). Major element plot between BCM and B2 (A-1); BC and BCR (A-2); BCM and BCR (A-3). Multivariate Euclidean distance calculated using standardized trace element concentrations (B). Trace element plot between BCM and B2 (B-2); BC and BCR (B-2); BCM and BCR (B-3).

Similarities among the three brines were calculated using the multivariate Euclidean distance based on the concentrations of major ions (in g L^−1^; Fig. 2-A) and standardized trace elements (Fig. 2-B). The uncertainty of each Euclidean distance was estimated by propagating the analytical errors of the chemical measurements.

Indicator Species Analysis, which includes the calculation of Indicator Value (IV), was conducted using the R package “indicspecies” (De Cáceres et al., 2010).

Brine samples were analyzed to investigate the relationship between OTUs and their habitats using the multinomial species classification method (CLAM) in the vegan package and the clamtest function in R (Chazdon et al., 2011; Pedrinho et al., 2020). The CLAM method categorizes OTUs into generalists (taxa that thrive across diverse environmental conditions and resources), specialists (taxa that flourish only in limited environmental settings requiring specific resources), or those considered too rare to be classified.

Correlations between abiotic parameters and the most abundant bacterial and fungal genera (relative abundance > 1 %) were calculated using the Pearson correlation coefficient with the r package “Hmisc” (Harrell, 2024). Only the abiotic parameters that were found simultaneously in the three brines were considered for the analysis. Only the correlations reporting a p-value < 0.05 were considered.

## 3 Results

### 3.1 Chemical characteristics of the brines

The *in-situ* brines exhibit different salinity, pH, and gas abundance. B2 and BCM show the highest salinity (>70 psu) and medium/high gas abundance, while BCR has a lower salinity (56.84 psu) and no detectable gas. B2 additionally displays the highest pH (7.42), whereas BCR and BCM have similar pH values (6.83 and 6.34, respectively).

Chemical data (Forte et al., 2024; Guglielmin et al., 2023) indicate that the three brines (B2, BCM, and BCR) share a common marine origin, as also demonstrated by Forte et al. (2024). Cryo-concentration processes enhanced their salinity, resulting in a proportional depletion of sodium sulphate compared to seawater (Guglielmin et al., 2023). This cryogenic fractionation was more pronounced in B2 and BCM, while the lower salinity of BCR can be attributed to dilution through greater interaction with the less saline BCG glacier ice. Consequently, in terms of solute concentrations, B2 is more similar to BCM than to BCR.

Here, the Euclidean multivariate distance (see Fig. 2-A) was calculated from the concentration values of all measured elements to estimate these similarities. B2 and BCM showed greater similarity (i.e., lower Euclidean distance; d = 30 ± 7), supported by their higher correlation in major ions (r² = 0.938; see Fig. 2-A-1).

The trace element analyses shown in Fig. 2-B evidenced the separation between the brines B2 and BCM (i.e., Euclidean distance; d = 6.12 ± 0,52) while highlighting the proximity of B2 with BCR with their lower standardized multivariate Euclidean distance (*d* = 3.66 ± 0.63) and a stronger trace element correlation (r² = 0.874).

### 3.2 Taxonomic composition of the microbial community of the brines

**Fig. 3:**
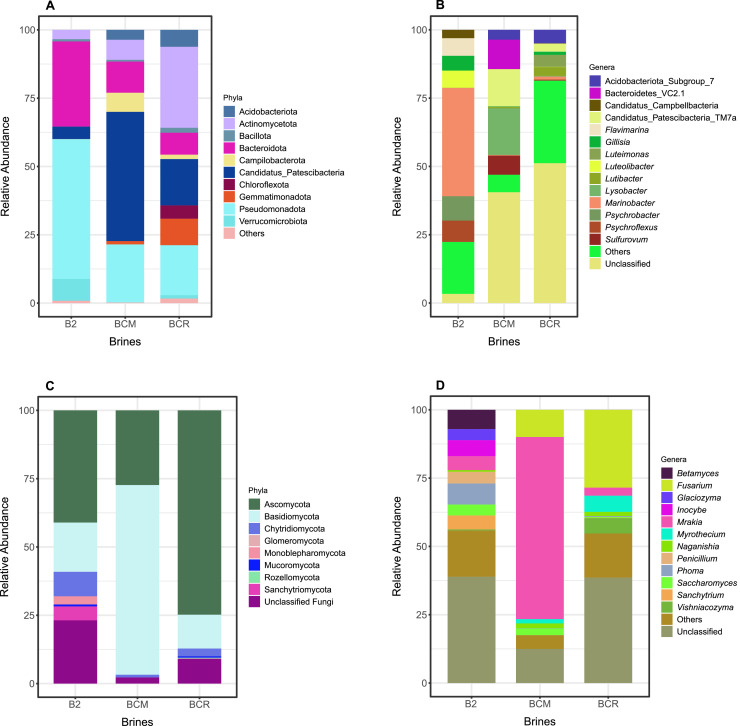
Microbial taxonomy composition of the three brines. A: relative abundance of bacterial phyla. B: relative abundance of bacterial genera. C: relative abundance of fungal phyla. D: relative abundance of fungal genera. Only taxa reporting a relative abundance > 1 % are reported.

At the phylum level, the results showed Pseudomonadota as the most represented phylum in B2 (51.28 %) and BCM (21.36 %), while its abundance dropped to 18.24 % in BCR (Fig. 3A). Among Pseudomonadota, Alpha- and Gammaproteobacteria were retrieved, with the Gamma- representing the majority, showing percentages of 50.92 %, 21.18 %, and 15.63 % for B2, BCM, and BCR, respectively. In comparison, Alphaproteobacteria showed very low abundance values, with the only relevant percentage in sample BCR (2.61 %) (Figure S1). Bacteroidota was another significant group, with high abundance in B2 (31.13 %) and BCM (11.47 %) but low in BCR (8.01 %). *Candidatus* Patescibacteria showed remarkable abundance in BCM (47.1 %), followed by BCR (16.9 %) and B2 (4.54 %). Actinomycetota was more represented in BCR (29.71 %) than in BCM (7.35 %) and B2 (3.38 %). Other phyla, such as Campylobacterota and Acidobacteriota, showed a significant percentage even if only in one of the analysed samples (e.g., Campylobacterota 7.02 % in BCM; Acidobacteriota 6.19 % in BCR and 3.62 % in BCM) (Fig. 3A). At the genus level, *Marinobacter* spp. and *Psychrobacter* spp. (belonging to phylum Pseudomonadota) dominated in B2, with 39.58 % and 9.04 %, respectively. B2 also showed relevant percentages of *Flavimarina* spp. (6.41 %) and *Gillisia* spp. (5.32 %) (phylum Bacteroidota). In BCM, *Saccharimonadales* spp. (clade *Patescibacteria*) was the most represented genus (29.48 %). Also, *Sulfurovum* spp. and *Lysobacter* spp. (belonging to the phyla Campylobacterota and Pseudomonadota, respectively) were highly abundant, with percentages of 7.02 % and 17.16 %, respectively. In BCR, a predominance of *Saccharimonadales* spp. (clade Patescibacteria) was observed (10.33 %). *Luteimonas* spp. (phylum Pseudomonadota) and *Sulfurimonas* spp. (phylum Campylobacterota) were also retrieved in BCR with significant percentages (4.32 % and 1.44 %, respectively) (Fig. 3B).

Ascomycota was the most abundant fungal phylum in B2 and BCR samples (relative abundance of 41 and 75 %, respectively). The other dominant fungal phyla were Basidiomycota and Chytridiomycota in B2. On the other hand, BCM was dominated by Basidiomycota and Ascomycota (relative abundance of 69 and 27 %, respectively) (Fig. 3C). Taxa unidentified at the genus level constituted the majority of the fungal community in B2 (38.89 %) and BCR (38.56 %), whereas their relative abundance was markedly lower in BCM (12.46 %). Both yeast and filamentous fungal forms were found. *Fusarium* spp. was not found in B2, was present at moderate levels in BCM (10.02 %), and was abundant in BCR (28.49 %). *Mrakia* spp. dominated BCM (66.59 %), while its occurrence in B2 (5.10 %) and BCR (2.94 %) was significantly lower. *Phoma* spp. was fairly high in B2 (7.72 %), but sparse in BCM (0.12 %) and BCR (0.32 %). *Saccharomyces* spp. was more common in B2 (4.00 %) and BCM (2.41 %), but much less so in BCR (0.31 %). *Sanchytrium* spp., *Betamyces* spp., and *Inocybe* spp. were found in B2 but were missing in BCM and BCR, while *Myrothecium* spp. and *Vishniacozyma* spp. were detected in BCR, with fewer instances in BCM and not present in B2 (Fig. 3D). Fungal taxonomy at the class, order, and family levels is reported in Figure S2.

### 3.3 Microbial diversity of the brines

**Fig. 4:**
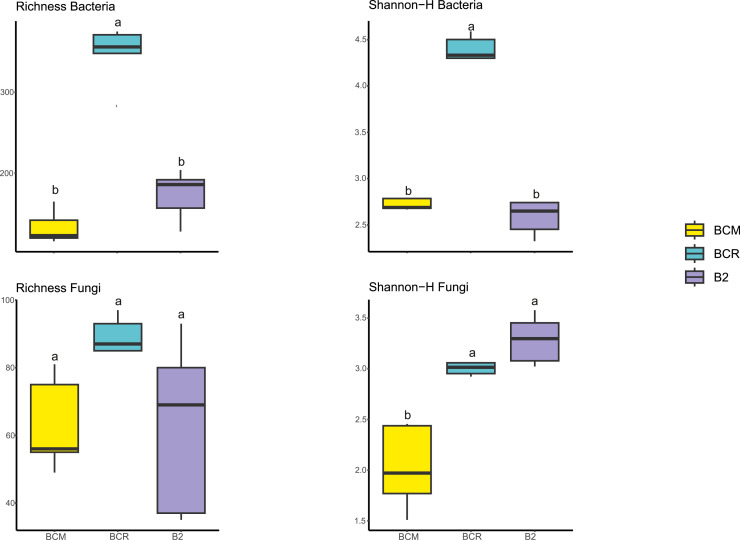
Bacterial and fungal alpha-diversity was calculated with richness and Shannon-H indexes. Significant (*p* < 0.05) differences among the brines are highlighted with different letters. Box plots represent the distribution of values across samples. For each brine, five replicates were analyzed.

**Fig. 5:**
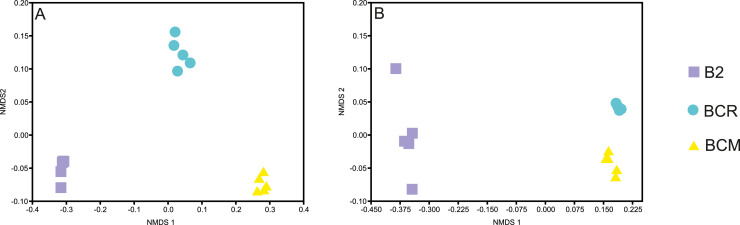
NMDS ordination reporting the spatial distribution of bacterial (A) and fungal (B) communities of the brines. Significant (*p* < 0.05) differences are reported in Table S1. For each brine, five replicates were analyzed.

The comparison of the bacterial community structure of the three brine samples revealed remarkable differences. Alpha-diversity, measured with the Shannon-H index, showed a value > 2 and comparable for samples B2 and BCM, while the highest diversity was found in the BCR sample with H' > 4 (Fig. 4). NMDS analysis showed a clear separation of the brines (Fig. 4a), indicating distinct microbial community compositions (Fig. 5A). The PERMANOVA test confirmed the existence of significant (*p* < 0.05) differences among the three bacterial communities found in B2, BCM, and BCR (Table S1). In contrast, the richness of fungal communities did not show significant (*p* < 0.05) differences among the brines, while significant (*p* < 0.05) differences were found by alpha-diversity measured with the Shannon-H index, with the highest value also found in BCM (Fig. 4). NMDS ordination for fungi revealed a clear separation among the three brines. PERMANOVA analysis also highlighted significant differences among them (*p* < 0.05) (Fig. 5B and Table S1). Bacterial communities showed generally low Jaccard similarities among brines, ranging from 0.09 to 0.22 (Table S1). The highest similarity was observed between BCM and BCR (Jaccard = 0.22), which shared 155 OTUs. Pairs involving B2 were more distinct, with lower similarity and fewer shared OTUs (BCM–B2: Jaccard = 0.09, 48 shared OTUs; BCR–B2: Jaccard = 0.11, 92 shared OTUs). RC values were very high and positive for all pairs (BCM–BCR: RC = 0.996; BCM–B2 and BCR–B2: RC = 1), indicating that bacterial structural differences among all three brines are much greater than expected by chance given sample richness and OTU occurrence frequencies (Table S1).

The structure of fungal communities exhibited a partly different pattern. BCM and BCR showed a Jaccard similarity of 0.42 and 101 shared OTUs. In contrast, both pairs involving B2 showed very low similarity (BCM–B2: Jaccard = 0.07, 20 shared OTUs; BCR–B2: Jaccard = 0.07, 23 shared OTUs). Consistently, the RC value for BCM–BCR was strongly negative (RC = –0.998), indicating fungal communities that are more similar than expected under the null model. In contrast, RC equal to 1 for both pairs involving B2, showing that the structure of fungal community in B2 is significantly (*p* < 0.05) different from those in BCM and BCR than expected by chance (Table S1).

### 3.4 Indicator taxa of bacterial and fungal communities in brines

The indicator taxa of the different brines were identified for bacteria and fungi at the family level, considering their highest Indicator Value (IV).

The bacterial indicator taxa in B2 included the families Moraxellaceae and Pseudomonadaceae (phylum Pseudomonadota), the families Cyclobacteriaceae and Flavobacteriaceae (phylum Bacteroidota), and the family Microbacteriaceae (phylum Actinomycetota), all exhibiting an IV of 0.99. In BCM, the bacterial indicator taxa were members of the families Xanthomonadaceae (phylum Pseudomonadota) and Saccharimonadaceae (phylum *Candidatus* Saccharimonadota), with IVs of 0.99 and 0.98, respectively. BCR showed the highest number of bacterial indicator taxa, namely members of the families Rhizobiaceae, Xanthomonadaceae (phylum Pseudomonadota), Saccharimonadaceae (phylum *Candidatus* Saccharimonadota), Caldilineaceae (phylum Chloroflexota), Flavobacteriaceae and Sphingobacteriaceae (phylum Bacteroidota), and Nitriliruptoraceae (phylum Actionomycota), all exhibiting IV ≥ 0.98 (Figure S3 and Table S2).

The fungal indicator taxa identified in B2 with the higher IV were the families Hyaloraphidiaceae (phylum Chytridiomycota), Aspergillaceae (phylum Ascomycota), and Sanchytriaceae (phylum Sanchytriomycota) (IV = 0.97, 0.94, and 0.92, respectively). The fungal indicator taxa identified in BCR were the families Stachybotryaceae, Chaetomiaceae, Nectriaceae, Microascaceae, and Pyronemataceae (phylum Ascomycota), exhibiting IV values of 0.95, 0.93, 0.92, 0.91, and 0.91, respectively. In BCM, only the family Mrakiaceae (phylum Basidiomycota) showed an IV > 0.9 (Figure S4 and Table S3).

### 3.5 Microbial species distribution in brine communities

**Fig. 6:**
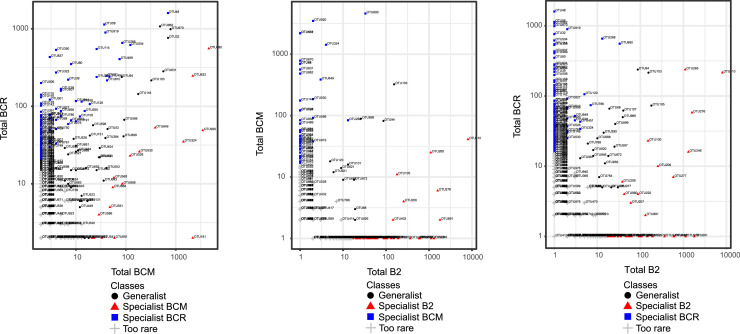
Classification of bacterial taxa as generalists or specialists using the CLAM statistical approach. The number and the proportion of generalist and specialist taxa are reported in Table S4. In the plots, the x- and y-axes represent the total number of reads assigned to each OTU in the brines.

The comparison of bacterial taxa distribution across the three brines revealed the existence of distinct generalist and specialist taxa distribution patterns. The comparison BCM *vs* BCR displayed a relatively higher proportion of generalist taxa (11.8 %) than the comparison B2 *vs* BCM (2.9 %) and B2 *vs* BCR (3.0 %). Specialist taxa were differently distributed across the brines. They were notably more abundant in the comparison B2 *vs* BCR (specialist taxa exclusive of BCR = 20.4 %) and BCM *vs* BCR (specialist taxa exclusive of BCR = 18.0 %). On the other hand, specialist taxa exclusive to B2 were more abundant when comparing B2 to BCM (11.5 %) and B2 to BCR (5.8 %). The percentage of taxa classified as too rare was variable the comparison B2 *vs* BCM exhibiting the highest proportion (74.5 %), followed by B2 *vs* BCR (70.9 %) and BCM *vs* BCR (67.3 %), highlighting potential differences in analyzed bacterial communities across brines (Fig. 6 and Table S4).

**Fig. 7:**
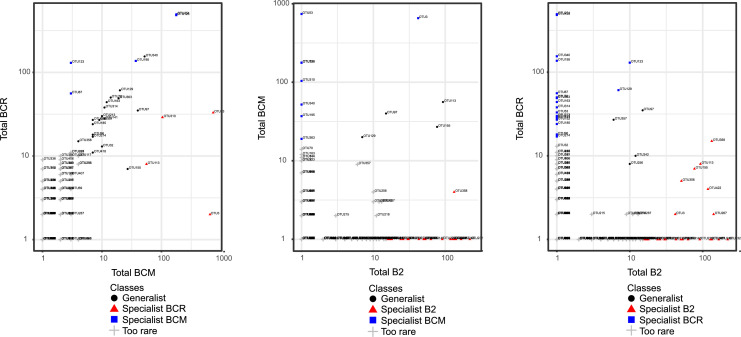
Classification of fungal taxa as generalists or specialists using the CLAM statistical approach. The number and the proportion of generalist and specialist taxa are reported in Table S5. In the plots, the x- and y-axes represent the total number of reads assigned to each OTU in the brines.

The comparison of fungal taxa distribution across the three brines (BCM, BCR, and B2) also revealed notable differences in the proportion of generalist, specialist, and rare taxa. Almost all fungal species of the comparison BCM *vs* BCR (93.7 %) were classified as too rare, while generalist taxa accounted for 4.2 %. The number of specialist taxa was low, with only 0.9 % exclusive to BCM and 1.2 % exclusive to BCR. Rare fungal taxa accounted for 91.8 % in the comparison BCM *vs* B2, while generalist species were 0.9 %. The proportion of specialists was 5.4 % in B2 and 1.9 % in BCM. When comparing BCR to B2, the rare fungal taxa accounted for 88.6 %, while generalists were 0.9 %. In B2, specialist taxa were 6.1 %, while in BCR, they were 4.4 %. (Fig. 7 and Table S5)

### 3.6 Correlation between microbial communities and the environmental parameters

Significant correlations (*p* < 0.05) were found between both bacterial and fungal genera and abiotic parameters. Among bacteria, the genera most frequently involved were *Candidatus* Patescibacteria TM7a, *Flavimarina, Gillisia, Marinobacter*, and *Psychroflexus*, and the abiotic parameters most frequently observed were B, Ca, Cl, Cu, Fe, Mn, U, and V (Figure S5 and Table S6).

Considering fungal communities, the genera most frequently involved were *Betamyces, Fusarium, Glaciozyma, Myrothecium, Penicillium, Phoma*, and *Sanchytrium*, and the abiotic parameters mainly observed were Ag, Cd, Cl, K, Na, Ni, and total nitrogen. Notably, *Mrakia* displayed a unique correlation profile with As, Co, Cr, Cu, P, Pb, and Se (Figure S6 and Table S7).

## 4 Discussion

The distinct chemical profile of the three brines suggests different hydrological flow paths. In particular, the Euclidean multivariate distance, calculated from the concentration values of all measured elements and used to estimate these similarities, showed that B2 and BCM are more similar (i.e., lower Euclidean distance; d = 30 ± 7), due to their higher correlation in major ions (r² = 0.938; see Fig. 1-A-1). However, this strong correlation primarily reflects their elevated major-ion concentrations, suggesting that both brines experienced similarly intense cryo-concentration processes. The more detailed analysis of trace elements revealed that lithogenic elements are more abundant in BCM than in the other brines, indicating stronger interaction with the frozen ground. Hence, from a trace-element perspective, BCM differed from the other brines (Forte et al., 2024), due to a lack of interconnection with B2. Conversely, despite their differing salinities, B2 and BCR appear to be interconnected, because of a lower standardized multivariate Euclidean distance (d = 3.66 ± 0.63) and a stronger trace-element correlation (r² = 0.874), which corroborates their similarity. Therefore, a probable independent pattern exists through the saline ice of RGP. At the same time, B2 and BCR appear to be connected, and through the BCG glacier, this connection led to a reduced ion concentration in BCR relative to B2 (Forte et al., 2024). This is reflected in an interpretation where BCM is isolated from glacial dilution influences, while B2 and BCR are connected via subsurface flow affected by meltwater input. This resembles the intra-system variation observed between TF4 and TF5 brines (Papale et al., 2019), which were chemically and microbiologically distinct despite being separated by only 12 cm of ice. This chemical situation creates unique ecosystems that are reflected in assembling distinct microbial communities. In particular, the microbial communities inhabiting the Boulder Clay brines under study (B2, BCR, and BCM) exhibited notable differences, emphasizing the geochemical specificity of each site (Santini et al., 2022). This is confirmed by NMDS and PERMANOVA analyses, which reported different bacterial and fungal communities in the three brines analysed, and is also in line with previous studies on Antarctic brines, where microbial communities were closely related to the physicochemical site-specific profiles (Papale et al., 2019).

The brine B2, characterized by the highest salinity (>70 psu), neutral pH (7.42), and elevated gas abundance, exhibited a community dominated by the class Gammaproteobacteria (50.92 %), including the key taxa *Marinobacter* (39.58 %) and *Psychrobacter* (9.04 %). These genera, well known for their halotolerance and cold adaptation (Ma et al., 2010), suggest that the salinity of B2 creates a selective pressure shaping the structure of the bacterial community. Additionally, the genera *Flavimarina* and *Gillisia* (phylum Bacteroidota) indicate a possible availability of organic labile matter (Nigro et al., 2020). This structure suggests the possible marine origin of brines, as both genera *Marinobacter* and *Psychrobacter* (phylum Pseudomonadota) are widely reported from marine and sea-ice environments, including polar waters. Regarding the fungal community, the abiotic conditions of the B2 brine were associated with the families Aspergillaceae (phylum Ascomycota) and Sanchytriaceae (phylum Sanchytriomycota) and with a percentage of specialist taxa (5.4 %), indicating a niche-adapted assembly, probably shaped by selective pressure from cryo-concentration and chemical stress (Barone et al., 2019). According to the extreme conditions, B2 also showed a significant proportion of unclassified fungi (∼39 %), suggesting that the extreme habitats under study can be considered a hotspot for potentially novel taxa (Barone et al., 2019; Mircea et al., 2024) or undersampled lineages. This pattern partially diverges from that observed in the Tarn Flat brines where fungal communities were dominated by Basidiomycota (especially by *Leucosporidium* spp. and *Naganishia* spp.) and showed only ∼4 % of unclassified taxa (Borruso et al., 2018). The structure of microbial communities herein reported supports the hypothesis that marine-origin brines undergo progressive cryo-concentration, as also hypothesized for other Antarctic systems (Guglielmin et al., 2023).

The brine BCM, which showed similarities with B2 in terms of major ion composition (Euclidean distance *d* = 30 ± 7; r² = 0.938), differed significantly in trace elements, likely due to enhanced interaction with the frozen till (Forte et al., 2024). This geochemical characteristic corresponded to a distinct microbial profile. Considering bacterial communities, BCM was dominated by *Candidatus* Patescibacteria (47.1 %), with the order Saccharimonadales (29.48 %). Patescibacteria, a phylum of ultra-small cells, are genome-reduced bacteria often implicated in symbiotic or syntrophic lifestyles (Smedile et al., 2024). The presence of the genera *Lysobacter* (17.16 %) and *Sulfurovum* (7.02 %) (phyla Gammaproteobacteria and Campylobacterota, respectively) suggests microbial sulfur cycling and potential mineral interactions, which are consistent with the trace-metal-enriched, low-pH (6.34) environment (Huang et al., 2023). This highly specialized and reduced bacterial profile, especially the higher presence of *Candidatus* Patescibacteria, parallels the uniqueness reported in the endoglacial brine (Guglielmin et al., 2023). Furthermore, Patescibacteria are generally reported as a permafrost-resident bacterial taxon (Alekseev et al., 2020; Frey et al., 2016; Hu et al., 2016), which supports the possible influence of the permafrost on this brine, as evidenced by the chemical results. Considering fungal communities, the brine was dominated by the genus *Mrakia* (phylum Basidiomycota). This yeast genus is one of the dominant and adaptable fungal taxa in cold environments (Turchetti et al., 2025). It could significantly impact on biogeochemical cycles in cold ecosystems, primarily through the release of several extracellular cold-active hydrolytic enzymes. This enables *Mrakia* species to hydrolyse various organic polymers over a wide temperature range (Turchetti et al., 2025). The dominance of *Mrakia* spp. in BCM represents a clear divergence from the profiles observed in other Antarctic brines. For instance, neither *Mrakia* nor *Fusarium* was detected in the endoglacial brine described by Guglielmin et al. (2023). While the brine shared a similar high salinity with BCM, its fungal community was dominated by filamentous Ascomycetes, such as *Cladosporium* and *Penicillium*, and lacked *Fusarium*, despite the overall prevalence of Ascomycota. The lack of *Mrakia* likely reflects different pH and mineral conditions compared to BCM. This divergence suggests that the distinct ecological niche of BCM may be related to its mineral-rich environment and slightly more acidic conditions compared to the other brines.

The brine BCR exhibited the lowest salinity (56.84 psu), slightly acidic pH (6.83), no detectable presence of gas and the highest bacterial diversity (Shannon-*H* > 4). It was dominated by Actinobacteria (29.71 %), followed by *Candidatus* Patescibacteria (16.9 %) and Gammaproteobacteria (phylum Pseudomonadota) (15.63 %). The presence of the genera *Luteimonas* and *Sulfurimonas* (phyla Pseudomonadota and Campylobacterota), known for their roles in carbon and sulfur cycling (Zhao et al., 2023), may reflect the influence of glacier-derived dilution, which likely reduced salinity and introduced substrates favoring their metabolic activities. Notably, indicator taxa in BCR included a few families like Caldilineaceae (phylum Chloroflexota), Rhizobiaceae (phylum Pseudomonadota), and Sphingobacteriaceae (phylum Bacteroidota) (IV ≥ 0.99), which are often associated with freshwater or soil habitats (Trivedi et al., 2020). These taxonomic patterns are similar to those observed in glacial meltwater environments and proglacial lakes (Peoples et al., 2025), as well as in cryoconite holes, which serve as microbial hotspots on glacier surfaces, where organic and inorganic debris are accumulated and subsequently colonized by microorganisms from ice, the atmosphere, and nearby soils. These communities often include abundant Actinomycetota, Bacteroidota, and Pseudomonadota (Cameron et al., 2012). The presence of these groups in BCR thus indicates an active interaction between the brine and the nearby glacial-terrestrial environment, where melt processes introduce fresh inputs.

Fungal communities of BCR also included terrestrial-associated families such as Stachybotryaceae (Li and Jiang, 2011; Samarakoon et al., 2021; Tennakoon et al., 2021), and Nectriaceae (phylum Ascomycota), which are generally less tolerant to high salinity. Their presence in BCR is ecologically consistent with the lower salinity conditions observed at this site, likely resulting from glacial meltwater dilution. This aligns with previous findings showing that the family Nectriaceae is significantly less abundant in highly saline-alkaline environments and is typically replaced by more salt-tolerant taxa (Rath and Rousk, 2015). This interpretation is further supported by comparison with the Boulder Clay brines described in Sannino et al. (2020), where fungal genera such as *Cladosporium* (phylum Ascomycota), *Dioszegia*, and *Naganishia* (phylum Basidiomycota) dominated across multiple brines, while the genus *Fusarium* (phylum Ascomycota, found to be highly abundant in BCR in the present study) was not detected. This discrepancy underscores the spatial heterogeneity within the same region and suggests that the soil- or glacier-derived fungal taxa found in BCR are likely the result of local hydrological dilution and ecotonal positioning.

The integration of abundance profiles, indicator Species Analysis (IV), and CLAM specialization offers a multidimensional perspective on microbial relationships with Boulder Clay brines. Although these approaches rely on different principles, they converged for a subset of taxa that are both numerically dominant and strongly confined to a single brine. For instance, *Marinobacter* in B2 was the most abundant taxon, exhibited a very high IV, and was classified as a B2 specialist by CLAM. Similarly, Saccharimonadales dominated BCM, showed an IV of 0.99, and was identified as a BCM specialist. In the brine BCR, the families Rhizobiaceae and Caldilineaceae also displayed IV ≥ 0.99 and were classified as specialists. Fungal communities followed the same pattern: Aspergillaceae in B2 and Mrakiaceae in BCM were abundant and acted as indicator taxa (IV > 0.90) and specialists. Meanwhile, the families Stachybotryaceae and Nectriaceae in BCR also satisfied all three criteria.

Many taxa emerged as indicators, but not CLAM specialists because they occurred at low levels elsewhere, and most fungal OTUs (>80 %) were too rare for CLAM thresholds. These discrepancies may reflect the complementarity of the methods: IV captures habitat fidelity even for rare taxa, whereas CLAM emphasizes exclusivity. Taken together, these results demonstrate that abundance, IV, and specialization reflect three non-redundant ecological dimensions: numerical dominance, habitat preference, and habitat exclusivity (Devictor et al., 2010). Concordance among metrics occurs only when a taxon is truly dominant and nearly absent elsewhere (Chazdon et al., 2011), whereas mismatches may reveal taxa that are either strong indicators but not specialists (Tsai et al., 2021), or rare but highly faithful to a single brine (Isola and Prenafeta-Boldú, 2025). Therefore combined use of these three approaches provides a more nuanced understanding of the structure of microbial communities in hypersaline Antarctic brines (Zucconi et al., 2025).

Both bacterial and fungal results support the hypothesis that BCR represents a transitional environment shaped by the influx of freshwater, where glacial meltwater dilutes salinity and introduces soil-derived taxa. This cryptic scenario shows the influence of sediment and glacial inputs (La Ferla et al., 2017), where local sedimentary conditions and water exchanges shaped the microbial profiles in Boulder Clay permafrost.

The results herein reported reveal significant microbial differences among the three brines, despite their close spatial proximity, highlighting the influence of localized environmental filters in shaping microbial communities. However, the Raup-Crick (RC) ecological index, together with Jaccard similarity, indicated that bacterial communities in all three brines are more dissimilar than expected under random assembly, consistently with strong environmental filtering rather than unrestricted dispersal. In contrast, fungal communities showed a tendency towards homogenization between BCM and BCR, whereas those in B2 remained distinct, suggesting taxon-specific patterns of connectivity and selection among the brines.

Significant correlations between bacterial and fungal genera and chemical parameters suggested that community composition could be related to ionic and nutrient gradients. Among bacteria, *Flavimarina* and *Gillisia* showed strong associations with K and Ca, while *Marinobacter* and *Psychrobacter* exhibited the highest correlations with total nitrogen. These patterns are consistent with previous studies demonstrating that macroelements and nutrient availability strongly affect the structure of microbial community in hypersaline environments (Liu et al., 2018; Podell et al., 2013). In addition, *Candidatus* Patescibacteria TM7a and *Psychroflexus* were frequently involved in correlations with trace elements such as Ag, Cd, and V, suggesting that ultra-small and halophilic taxa may exploit micronutrient niches in the studied brines.

Regarding fungi, *Penicillium* and *Sanchytrium* correlated with K and total nitrogen, confirming that nutrient availability acts as a selective filter for eukaryotic communities as well (Farouk et al., 2025; Howarth et al., 2021). Other genera, including *Betamyces* and *Phoma*, also displayed strong associations with K, Cd, and total nitrogen, indicating their ecological relevance in nutrient-rich microhabitats. Notably, the yeast genus *Mrakia* exhibited a unique correlation profile with some metals, reflecting its adaptation to mineral-rich niches in cold environments (Turchetti et al., 2025). These findings reinforce the hypothesis that chemical variability, rather than spatial connectivity alone, may be the main driver of microbial differentiation in Antarctic brines (Borruso et al., 2018).

The structure of both bacterial and fungal communities responded to ecologically relevant variations in salinity, trace elements, and substrate context (Forte et al., 2024; Guglielmin et al., 2023), supporting the idea that even in extreme and tightly confined ecosystems, environmental heterogeneity can create unique microbial niches, leading to high community turnover and species specialization (Sannino et al., 2020; Santini et al., 2022). The Boulder Clay brines thus could be used as a model system to study how strong abiotic filters and microhabitat isolation affect microbial diversity in extreme environments (Azzaro et al., 2021; Barone et al., 2019).

## 5 Conclusions

The microbial distribution observed in the three brines highlights that both bacterial and fungal community structure is shaped by environmental variability. Each brine primarily act as a distinct ecological filter, favouring specific taxa based on local abiotic constraints. The differences of community structure are not simply a matter of variable abundance but reflect pronounced taxonomic turnover and habitat filtering, with most microbial taxa showing restricted occurrence across brines. This pattern is particularly evident in fungal communities, where the dominance of rare taxa and the scarcity of generalist taxa suggest selective conditions that limit taxonomic overlap. Jaccard and RC analyses confirm that the differences among communities are not the product of a simple random turnover. The structure of bacterial communities in all three brines are more dissimilar than expected by chance, indicating strong deterministic filtering. In contrast, fungal communities showed partial homogenisation between BCM and BCR and a strongly distinct structures in B2, indicating taxon-specific connectivity superimposed strong local selection. These results could also support the interpretation that BCM behaves as a geochemically isolated system, while B2 and BCR, could form a hydrological continuum affected by marine-derived brine progressively diluted by glacier meltwater

## Funding

This research was funded by Italian National Program for Antarctic Research (PNRA), grant numbers PNRA-PNRA16_00194 and PNRA18_00186.

## Declaration of competing interest

The authors declare that they have no known competing financial interests or personal relationships that could have appeared to influence the work reported in this paper.

## Appendix Supplementary materials

Image, application 1
