# Supplementary material for: Revealing unexplored bacterial and fungal variability in interconnected Antarctic brines
Source: Curr Res Microb Sci. 2025 Dec 18;10:100538. doi: 10.1016/j.crmicr.2025.100538 (PMC12811473; doi:10.1016/j.crmicr.2025.100538)
Supplement: Supplementary file 1 [file mmc1.pdf]

Revealing unexplored bacterial and fungal variability in interconnected Antarctic brines

Maria Papale, Ciro Sannino*, Dario Battistel, Gianmarco Mugnai, Luigimaria Borruso, Angelina Lo Giudice, Benedetta Turchetti, Maurizio Azzaro, Pietro Buzzini, Mauro Guglielmin

Figure S1. Bacterial Taxonomy composition in the three brines. A: relative abundance of bacterial classes. B: relative abundance of bacterial orders. C: relative abundance of bacterial families. Only taxa reporting a relative abundance > 1% are reported.

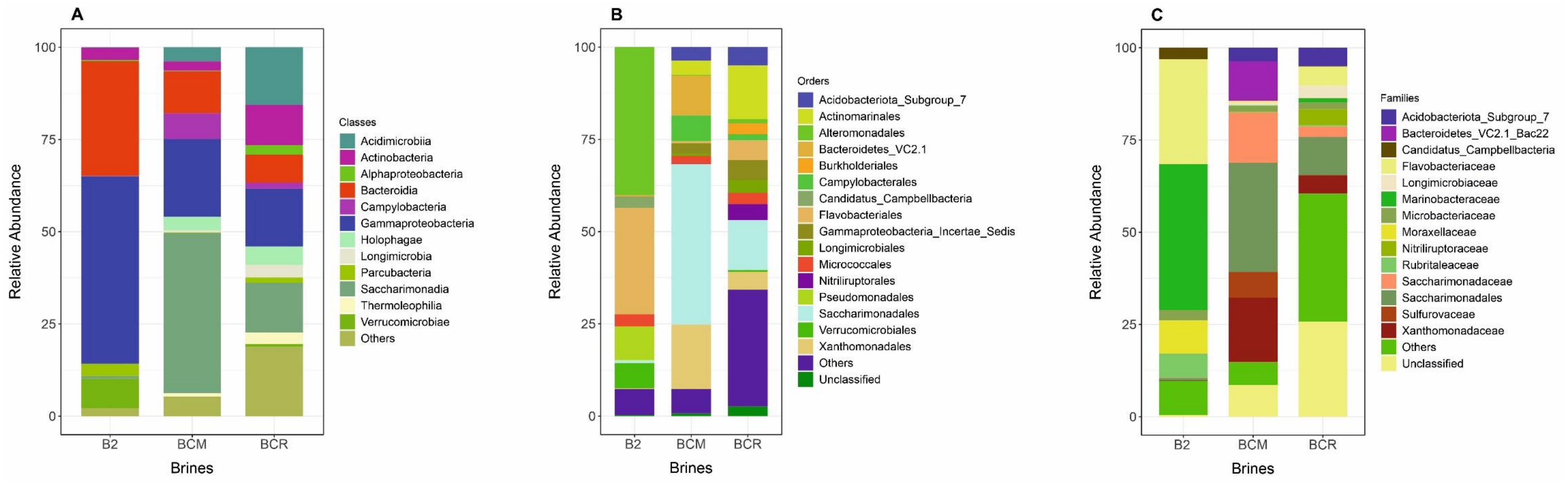

Figure S2. Fungal Taxonomy composition in the three brines. A: relative abundance of fungal classes. B: relative abundance of fungal orders. C: relative abundance of fungal families. Only taxa reporting a relative abundance > 1% are reported.

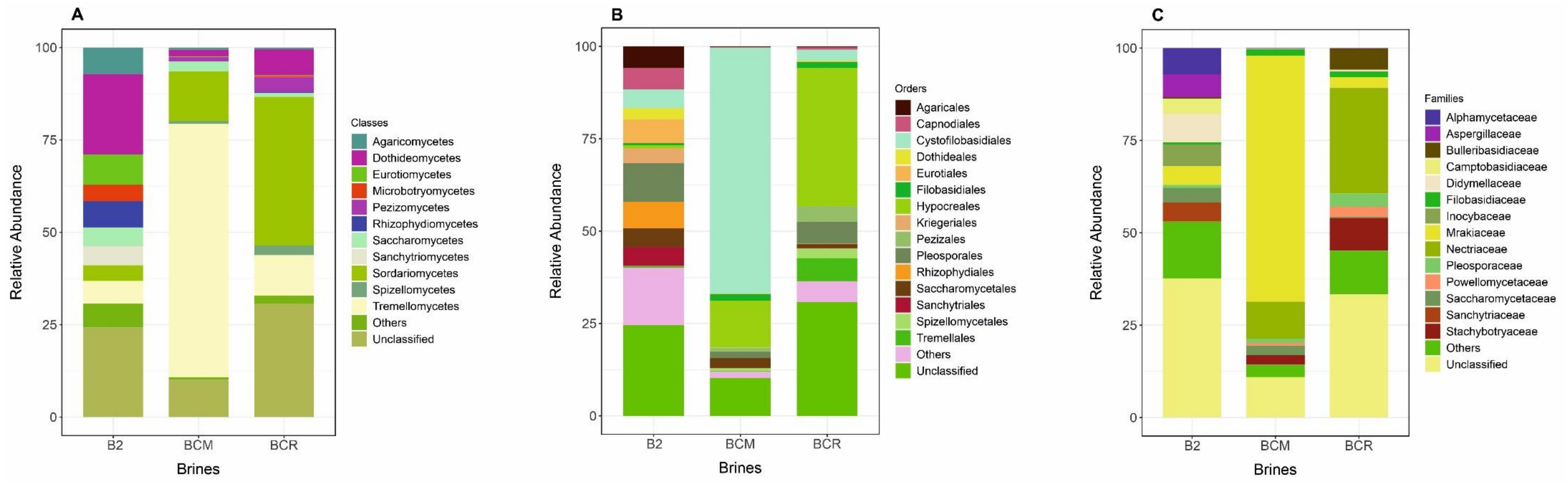

Figure S3. Graphical representation of the most representative bacterial families.

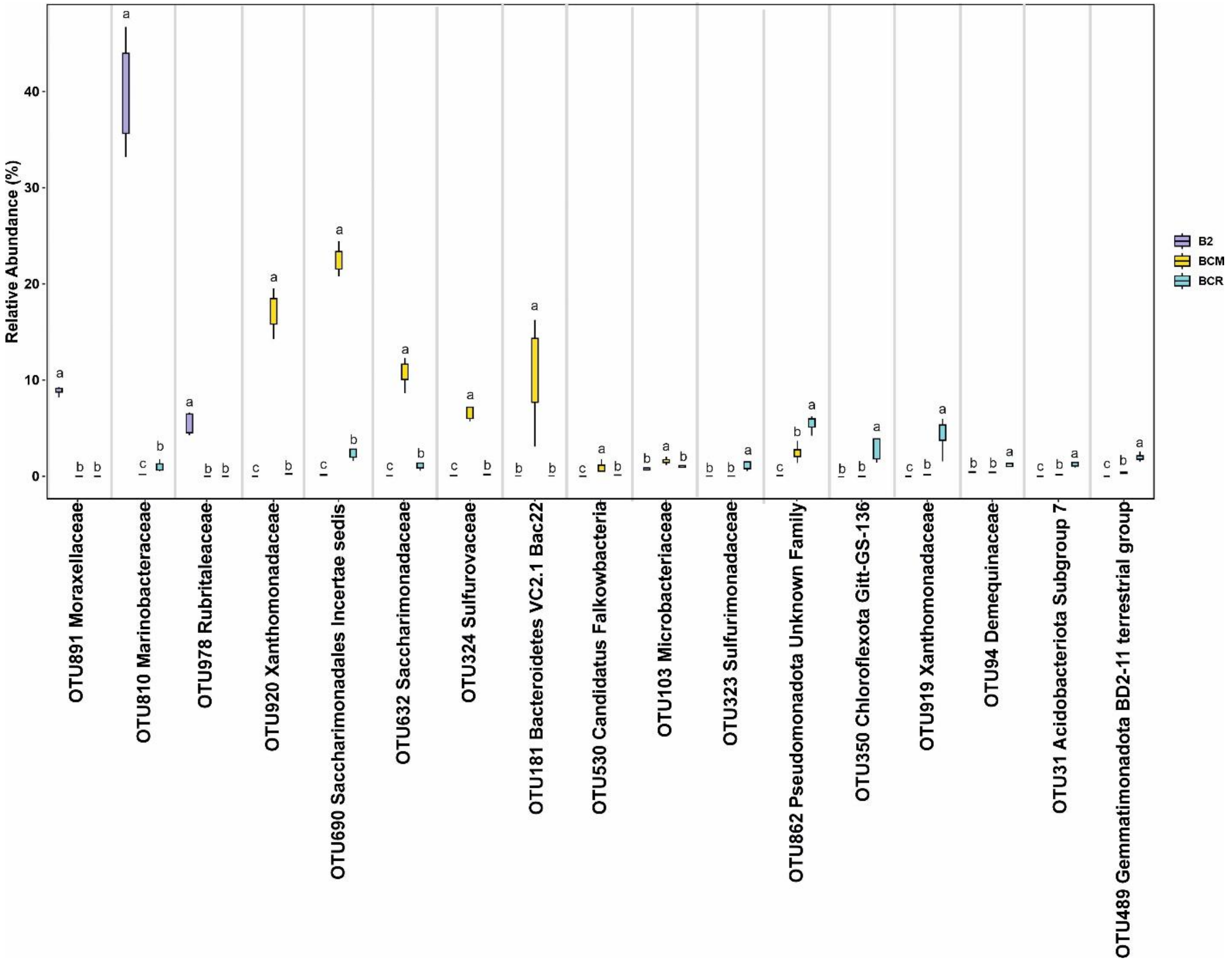

Figure S4. Graphical representation of the most representative fungal families.

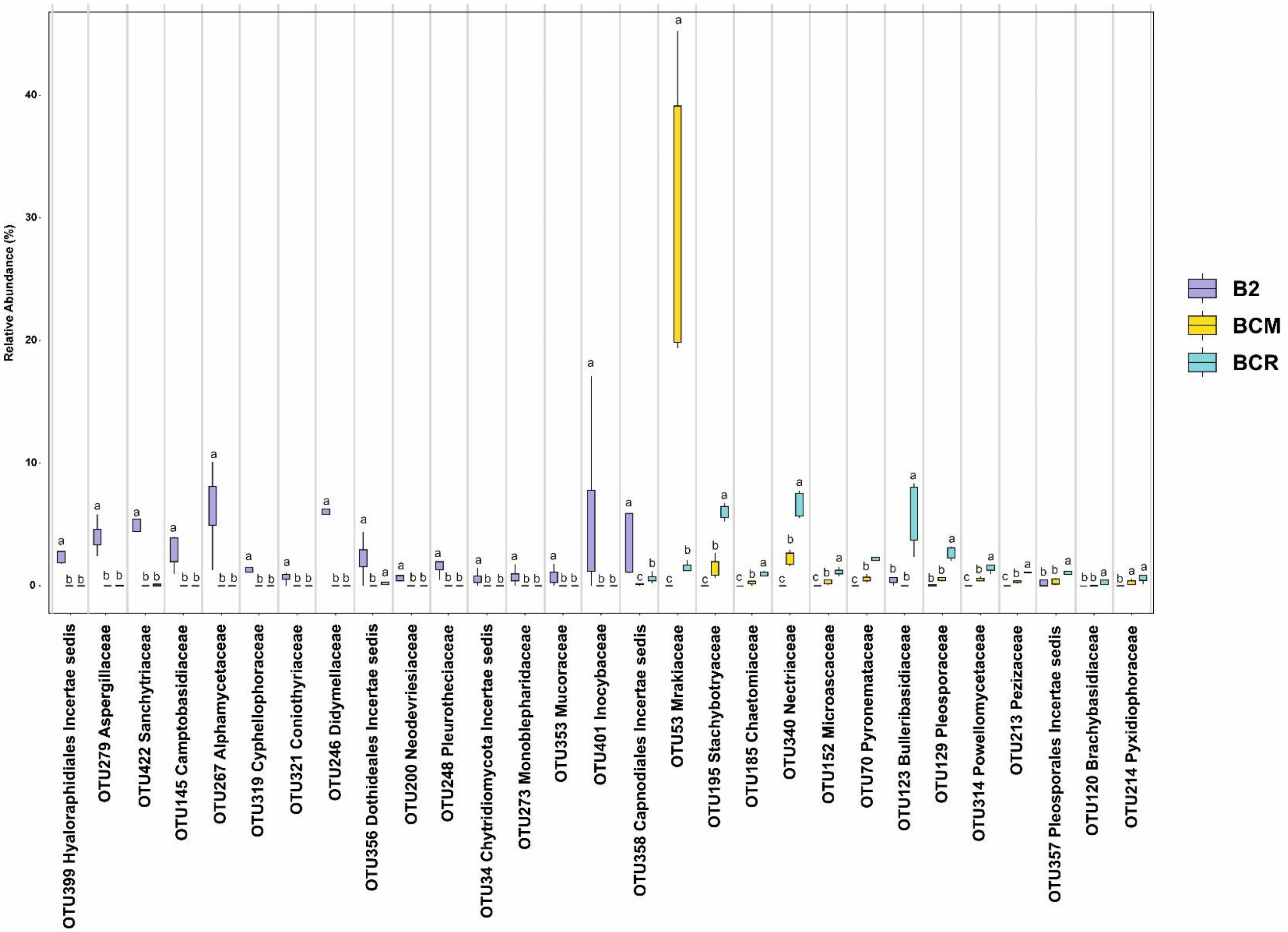

Figure S5. Correlations (calculated with the Pearson coefficient) among the most abundant bacterial genera (relative abundance > 1%) and abiotic parameters. The list of the significant ($p < 0.05$) correlations is reported in Table S6. EC: electrical conucibility; Cl: chlorine; SO4: sulfate; Na: Sodium; K: potassium; Ca: calcium; Mg: magnesium; Ctot: total carbon; Ntot: total nitrogen; Ag: silver; As: arsenic; B: boron; Ba: barium; Cd: cadmium; Co: cobalt; Cr: chromium; Cu: copper; Fe: iron; Mn: manganese; Ni: nickel; P: phosphorus; Pb: lead; Rb: rubidium; Se: selenium; U: uranium; V: vanadium.

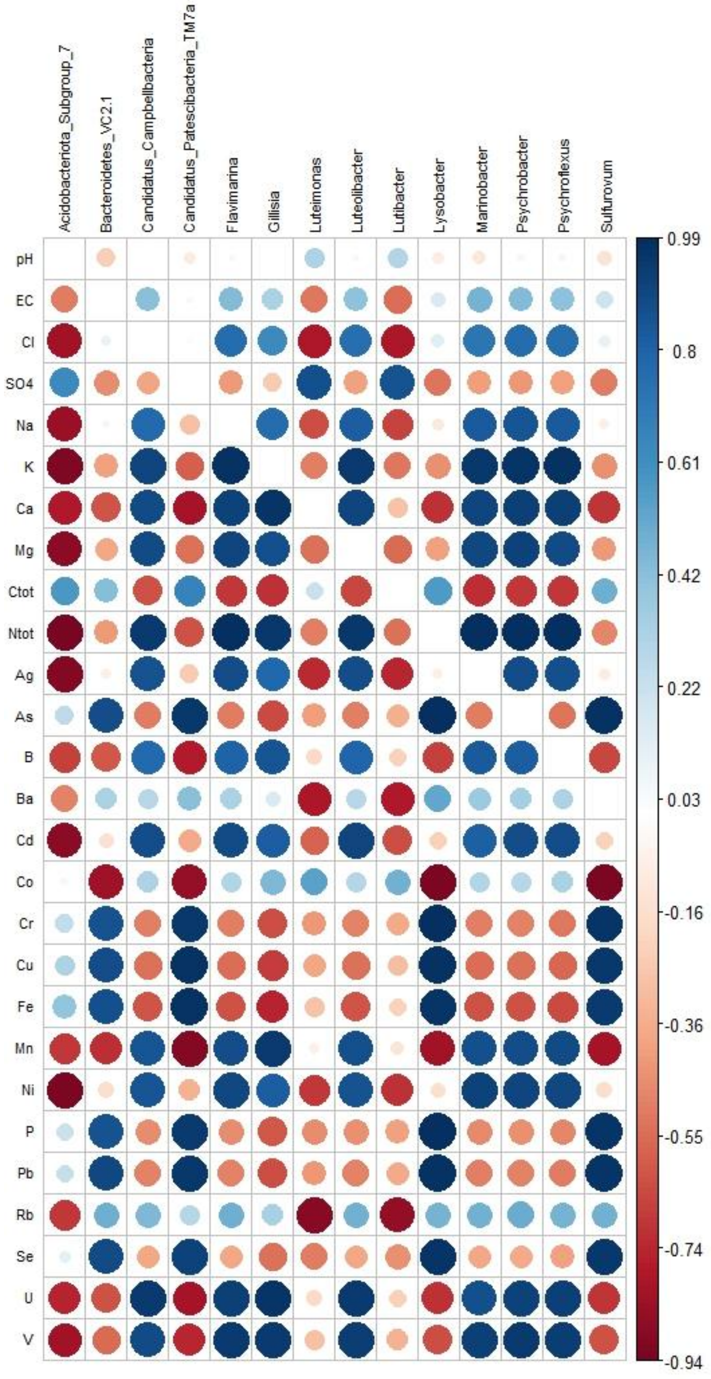

Figure S6. Correlations (calculated with the Pearson coefficient) among the most abundant fungal genera (relative abundance > 1%) and abiotic parameters. The list of the significant ($p < 0.05$) correlations is reported in Table S7. EC: electrical conucibility; Cl: chlorine; SO4: sulfate; Na: Sodium; K: potassium; Ca: calcium; Mg: magnesium; Ctot: total carbon; Ntot: total nitrogen; Ag: silver; As: arsenic; B: boron; Ba: barium; Cd: cadmium; Co: cobalt; Cr: chromium; Cu: copper; Fe: iron; Mn: manganese; Ni: nickel; P: phosphorus; Pb: lead; Rb: rubidium; Se: selenium; U: uranium; V: vanadium.

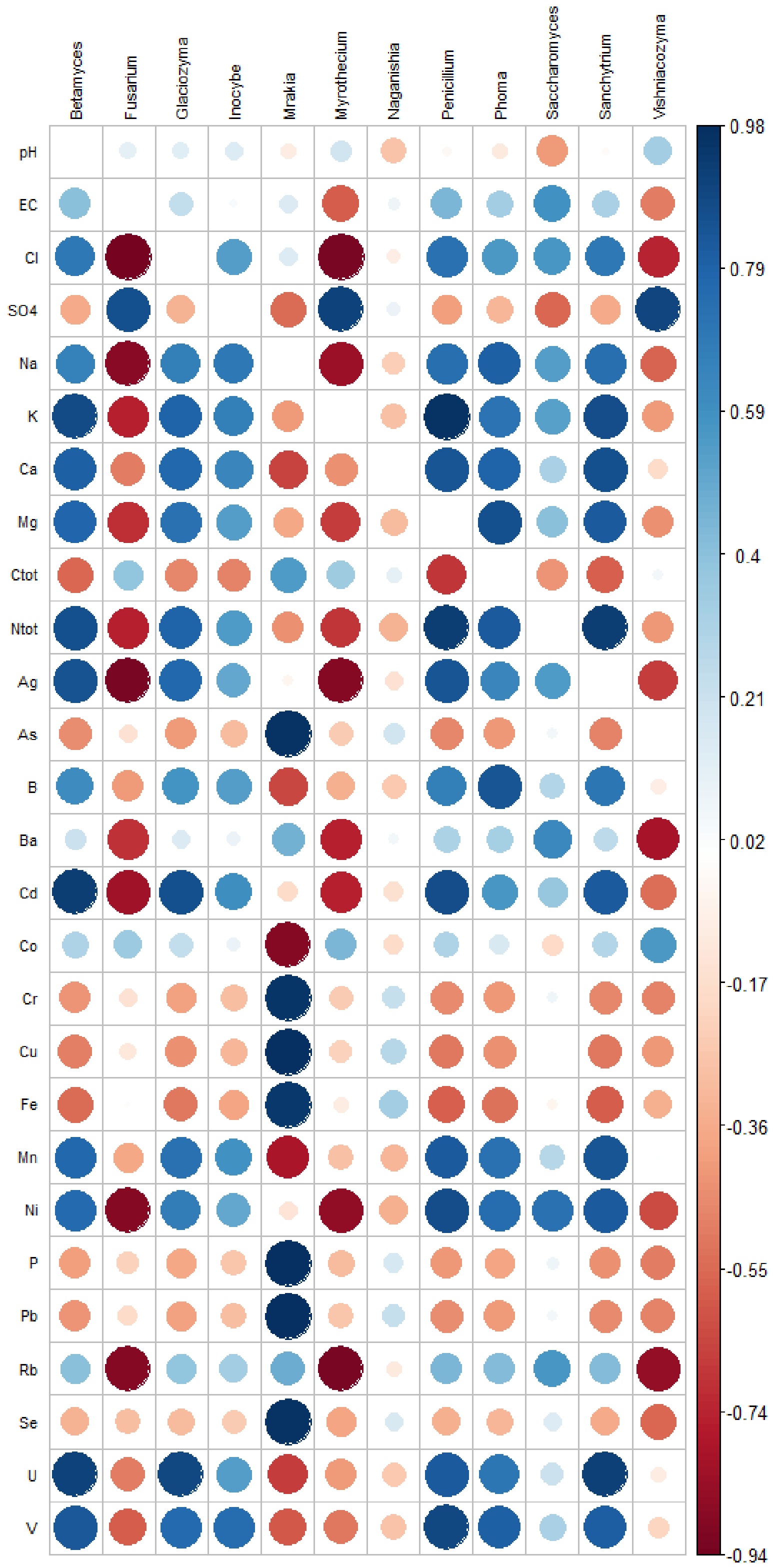

Table S1. PERMANOVA and Raup-Crick (RC) results showing significant differences ($p < 0.05$) in the composition of bacterial and fungal communities among brine samples.

	**PERMANOVA**				
**Bacteria**	**R2**	***p*-value**	**Jaccard**	**Raup-Crick**	**Shared OTUs**
BCM *vs*. BCR	0.915	0.001	0.222	0.996	155
BCM *vs*. B2	0.978	0.006	0.089	1	48
BCR *vs*. B2	0.953	0.007	0.109	1	92
**Fungi**	**R2**	***p*-value**			
BCM *vs*. BCR	0.719	0.006	0.419	-0.998	101
BCM *vs*. B2	0.635	0.014	0.067	1	20
BCR *vs*. B2	0.726	0.005	0.07	1	23

Table S2. List of the significant ($p < 0.05$) bacterial indicator species of the brines. IV, indicator value.

Brine	Kingdom	Phylum	Class	Order	Family	IV	p value	N OTU	Representative OTU
B2	Bacteria	Pseudomonadota	Gammaproteobacteria	Pseudomonadales	Moraxellaceae	0.999	0.0015	2	OTU891
B2	Bacteria	Bacteroidota	Bacteroidia	Flavobacteriales	Flavobacteriaceae	0.998	0.0015	13	OTU244
B2	Bacteria	Actinomycetota	Actinobacteria	Micrococcales	Microbacteriaceae	0.997	0.0015	3	OTU106
B2	Bacteria	Pseudomonadota	Gammaproteobacteria	Alteromonadales	Alteromonadaceae	0.995	0.0015	3	OTU803
B2	Bacteria	Bacteroidota	Bacteroidia	Cytophagales	Cyclobacteriaceae	0.995	0.0015	5	OTU207
B2	Bacteria	Pseudomonadota	Gammaproteobacteria	Oceanospirillales	Pseudohongiellaceae	0.995	0.0015	1	OTU881
B2	Bacteria	Pseudomonadota	Gammaproteobacteria	Pseudomonadales	Pseudomonadaceae	0.99	0.0015	1	OTU895
B2	Bacteria	Pseudomonadota	Gammaproteobacteria	Alteromonadales	Marinobacteraceae	0.987	0.0015	1	OTU810
B2	Bacteria	Bacteroidota	Bacteroidia	Flavobacteriales	Crocinitomicaceae	0.979	0.0015	1	OTU225
B2	Bacteria	Bacillota	Bacilli	Bacillales	Bacillaceae	0.978	0.0015	1	OTU402
B2	Bacteria	Verrucomicrobiota	Verrucomicrobiae	Verrucomicrobiales	Rubritaleaceae	0.978	0.0015	6	OTU978
B2	Bacteria	Bacteroidota	Bacteroidia	Sphingobacteriales	Bacteroidota_NS11-12_marine_group	0.976	0.0015	2	OTU296
B2	Bacteria	Pseudomonadota	Gammaproteobacteria	Xanthomonadales	Xanthomonadaceae	0.975	0.0015	2	OTU918
B2	Bacteria	Bacteroidota	Bacteroidia	Chitinophagales	Chitinophagaceae	0.967	0.0015	1	OTU186
B2	Bacteria	Patescibacteria	Parcubacteria	Candidatus_Campbellbacteria	Candidatus_Campbellbacteria	0.966	0.0015	2	OTU588
B2	Bacteria	Verrucomicrobiota	Verrucomicrobiae	Opitutales	Puniceicoccaceae	0.955	0.0015	1	OTU970
B2	Bacteria	Bacteroidota	Bacteroidia	Bacteroidales	Dysgonomonadaceae	0.954	0.0015	1	OTU175
B2	Bacteria	Bdellovibrionota	Bdellovibrionia	Bacteriovoracales	Bacteriovoracaceae	0.947	0.0015	1	OTU313
B2	Bacteria	Patescibacteria	Saccharimonadia	Saccharimonadales	Saccharimonadales_Incertae_sedis	0.947	0.0015	4	OTU664
B2	Bacteria	Planctomycetota	Planctomycetes	Pirellulales	Pirellulaceae	0.943	0.0015	4	OTU722
B2	Bacteria	Patescibacteria	Gracilibacteria	JGI_0000069-P22	Patescibacteria_JGI_0000069-P22	0.93	0.0015	1	OTU562
B2	Bacteria	Pseudomonadota	Gammaproteobacteria	Burkholderiales	Comamonadaceae	0.919	0.0015	4	OTU826
B2	Bacteria	Pseudomonadota	Gammaproteobacteria	Xanthomonadales	Rhodanobacteraceae	0.908	0.0015	2	OTU906
B2	Bacteria	Verrucomicrobiota	Verrucomicrobiae	Verrucomicrobiales	Verrucomicrobiota_DEV007	0.901	0.0015	1	OTU977
B2	Bacteria	Cyanobacteriota	Cyanobacteriia	Leptolyngbyales	Leptolyngbyaceae	0.885	0.0015	2	OTU367
B2	Bacteria	Pseudomonadota	Alphaproteobacteria	Acetobacterales	Acetobacteraceae	0.866	0.0015	1	OTU736
B2	Bacteria	Verrucomicrobiota	Verrucomicrobiae	Verrucomicrobiales	Verrucomicrobiaceae	0.866	0.0015	2	OTU992
B2	Bacteria	Cyanobacteriota	Cyanobacteriia	Cyanobacteriales	Phormidiaceae	0.863	0.0015	1	OTU365
B2	Bacteria	Bacteroidota	Bacteroidia	Cytophagales	Microscillaceae	0.853	0.0125	1	OTU220

B2	Bacteria	Bacteroidota	Bacteroidia	Chitinophagales	Saprospiraceae	0.831	0.0015	1	OTU198
B2	Bacteria	Patescibacteria	Saccharimonadia	Saccharimonadales	Saccharimonadaceae	0.83	0.0015	1	OTU634
B2	Bacteria	Verrucomicrobiota	Verrucomicrobiae	Chthoniobacterales	Terrimicrobiaceae	0.819	0.0015	1	OTU957
B2	Bacteria	Patescibacteria	Parcubacteria	Candidatus_Nomurabacteria	Candidatus_Nomurabacteria	0.799	0.0125	1	OTU606
B2	Bacteria	Pseudomonadota	Alphaproteobacteria	Rhodobacterales	Rhodobacteraceae	0.786	0.0125	3	OTU770
B2	Bacteria	Bacteroidota	Bacteroidia	Cytophagales	Spirosomaceae	0.774	0.0125	1	OTU221
B2	Bacteria	Bacteroidota	Bacteroidia	Cytophagales	Cytophagaceae	0.736	0.0125	1	OTU216
B2	Bacteria	Cyanobacteriota	Cyanobacteriia	Pseudanabaenales	Pseudanabaenaceae	0.733	0.0125	1	OTU372
B2	Bacteria	Desulfobacterota	Desulfuromonadia	Bradymonadales	Bradymonadaceae	0.707	0.012	1	OTU388
B2	Bacteria	Myxococcota	Polyangia	Polyangiales	Sandaracinaceae	0.707	0.0125	1	OTU516
B2	Bacteria	Pseudomonadota	Alphaproteobacteria	Caulobacterales	Caulobacteraceae	0.625	0.0441	1	OTU742
BCM	Bacteria	Pseudomonadota	Gammaproteobacteria	Xanthomonadales	Xanthomonadaceae	0.991	0.0011	2	OTU920
BCM	Bacteria	Patescibacteria	Saccharimonadia	Saccharimonadales	Saccharimonadales_Incertae_sedis	0.99	0.0011	6	OTU690
BCM	Bacteria	Patescibacteria	Saccharimonadia	Saccharimonadales	Saccharimonadaceae	0.98	0.0011	1	OTU632
BCM	Bacteria	Campilobacterota	Campylobacteria	Campylobacterales	Sulfurovaceae	0.972	0.0011	1	OTU324
BCM	Bacteria	Patescibacteria	Berkelbacteria	Berkelbacteria	Berkelbacteria__Incertae_sedis	0.971	0.0011	9	OTU551
BCM	Bacteria	Pseudomonadota	Gammaproteobacteria	Gammaproteobacteria_Incertae_Sedis	Pseudomonadota_Unknown_Family	0.963	0.0011	2	OTU858
BCM	Bacteria	Patescibacteria	Microgenomatia	Candidatus_Levybacteria	Candidatus_Levybacteria	0.923	0.0011	1	OTU563
BCM	Bacteria	Patescibacteria	Microgenomatia	Candidatus_Pacebacteria	Candidatus_Pacebacteria	0.916	0.0011	3	OTU566
BCM	Bacteria	Bacteroidota	Bacteroidia	Bacteroidetes_VC2.1_Bac22	Bacteroidetes_VC2.1_Bac22	0.878	0.0011	1	OTU181
BCM	Bacteria	Patescibacteria	ABY1	Candidatus_Kerfeldbacteria	Candidatus_Kerfeldbacteria	0.838	0.0011	1	OTU535
BCM	Bacteria	Pseudomonadota	Gammaproteobacteria	Thiomicrospirales	Thiomicrospiraceae	0.821	0.0027	1	OTU899
BCM	Bacteria	Patescibacteria	ABY1	Candidatus_Falkowbacteria	Candidatus_Falkowbacteria	0.815	0.0011	1	OTU530
BCM	Bacteria	Actinomycetota	Actinobacteria	Micrococcales	Microbacteriaceae	0.798	0.0019	1	OTU103
BCM	Bacteria	Bacillota	Clostridia	Clostridia_UCG-014	Clostridia_UCG-014	0.76	0.004	1	OTU449
BCM	Bacteria	Pseudomonadota	Gammaproteobacteria	MBAE14	Pseudomonadota_MBAE14	0.757	0.0114	1	OTU871
BCM	Bacteria	Pseudomonadota	Alphaproteobacteria	Sphingomonadales	Sphingomonadaceae	0.755	0.0114	1	OTU784
BCM	Bacteria	Actinomycetota	WCHB1-81	WCHB1-81	Actinomycetota_WCHB1-81	0.753	0.0097	1	OTU168
BCM	Bacteria	Bacillota	Bacilli	Lactobacillales	Streptococcaceae	0.664	0.0112	1	OTU416
BCM	Bacteria	Actinomycetota	Actinobacteria	Micrococcales	Intrasporangiaceae	0.634	0.0336	1	OTU97

BCM	Bacteria	Actinomycetota	Actinobacteria	Micrococcales	Micrococcaceae	0.616	0.0324	1	OTU108
BCR	Bacteria	Chloroflexota	Anaerolineae	Caldilineales	Caldilineaceae	0.993	0.0014	2	OTU328
BCR	Bacteria	Pseudomonadota	Alphaproteobacteria	Rhizobiales	Rhizobiaceae	0.993	0.0014	1	OTU751
BCR	Bacteria	Bacteroidota	Bacteroidia	Sphingobacteriales	Sphingobacteriaceae	0.993	0.0014	3	OTU301
BCR	Bacteria	Bacteroidota	Bacteroidia	Flavobacteriales	Flavobacteriaceae	0.99	0.0014	3	OTU243
BCR	Bacteria	Actinomycetota	Actinobacteria	Nitriliruptorales	Nitriliruptoraceae	0.99	0.0014	6	OTU110
BCR	Bacteria	Bacteroidota	Bacteroidia	Sphingobacteriales	Bacteroidota_NS11-12_marine_group	0.988	0.0014	3	OTU295
BCR	Bacteria	Gemmatimonadota	S0134_terrestrial_group	S0134_terrestrial_group	Gemmatimonadota_S0134_terrestrial_group	0.98	0.0014	3	OTU507
BCR	Bacteria	Bacillota	Desulfitobacteriia	Desulfitobacteriales	Desulfitobacteriaceae	0.977	0.0014	2	OTU473
BCR	Bacteria	Bacteroidota	Bacteroidia	Bacteroidales	Prolixibacteraceae	0.974	0.0014	1	OTU178
BCR	Bacteria	Actinomycetota	Thermoleophilia	Solirubrobacterales	Solirubrobacteraceae	0.973	0.0014	2	OTU162
BCR	Bacteria	Patescibacteria	Saccharimonadia	Saccharimonadales	Saccharimonadales_Incertae_sedis	0.97	0.0014	14	OTU656
BCR	Bacteria	Patescibacteria	Saccharimonadia	Saccharimonadales	Saccharimonadaceae	0.968	0.0001	1	OTU633
BCR	Bacteria	Gemmatimonadota	Gemmatimonadetes	Gemmatimonadales	Gemmatimonadaceae	0.967	0.0014	3	OTU499
BCR	Bacteria	Gemmatimonadota	Longimicrobia	Longimicrobiales	Longimicrobiaceae	0.966	0.0014	4	OTU500
BCR	Bacteria	Pseudomonadota	Alphaproteobacteria	Rhizobiales	Methyloligellaceae	0.964	0.0014	2	OTU750
BCR	Bacteria	Actinomycetota	Actinobacteria	Micrococcales	Demequinaceae	0.963	0.0014	1	OTU94
BCR	Bacteria	Bacteroidota	Rhodothermia	Rhodothermales	Rhodothermaceae	0.963	0.0014	1	OTU311
BCR	Bacteria	Bacillota	Clostridia	Peptostreptococcales-Tissierellales	Peptostreptococcales-Tissierellales _Incertae_sedis	0.961	0.0014	1	OTU466
BCR	Bacteria	Acidobacteriota	Holophagae	Subgroup_7	Acidobacteriota_Subgroup_7	0.961	0.0014	1	OTU31
BCR	Bacteria	Gemmatimonadota	BD2-11_terrestrial_group	BD2-11_terrestrial_group	Gemmatimonadota_BD2-11_terrestrial_group	0.96	0.0014	6	OTU489
BCR	Bacteria	Actinomycetota	Actinobacteria	PeM15	Actinomycetota_PeM15	0.96	0.0014	1	OTU117
BCR	Bacteria	Chloroflexota	Chloroflexia	Thermomicrobiales	Chloroflexota_AKYG1722	0.959	0.0014	1	OTU335
BCR	Bacteria	Verrucomicrobiota	Kiritimatiellae	WCHB1-41	Verrucomicrobiota_WCHB1-41	0.958	0.0014	3	OTU941
BCR	Bacteria	Patescibacteria	Parcubacteria	Candidatus_Kaiserbacteria	Candidatus_Kaiserbacteria	0.954	0.0014	2	OTU593
BCR	Bacteria	Pseudomonadota	Gammaproteobacteria	Salinisphaerales	Salinisphaeraceae	0.95	0.0014	1	OTU898
BCR	Bacteria	Pseudomonadota	Gammaproteobacteria	Burkholderiales	Comamonadaceae	0.948	0.0014	1	OTU827
BCR	Bacteria	Chloroflexota	JG30-KF-CM66	JG30-KF-CM66	Chloroflexota_JG30-KF-CM66	0.946	0.0014	3	OTU354
BCR	Bacteria	Actinomycetota	Acidimicrobiia	Microtrichales	Ilumatobacteraceae	0.944	0.0014	3	OTU67
BCR	Bacteria	Actinomycetota	Actinobacteria	Propionibacteriales	Nocardioidaceae	0.943	0.0014	3	OTU120

BCR	Bacteria	Bacillota	Symbiobacteriia	Symbiobacteriales	Symbiobacteraceae	0.942	0.0014	1	OTU478
BCR	Bacteria	Bacteroidota	Bacteroidia	Chitinophagales	Saprospiraceae	0.941	0.0014	3	OTU191
BCR	Bacteria	Actinomycetota	Actinobacteria	0319-7L14	Actinomycetota_0319-7L14	0.94	0.0014	1	OTU82
BCR	Bacteria	Bacillota	Clostridia	Clostridiales	Clostridiaceae	0.94	0.0014	2	OTU451
BCR	Bacteria	Patescibacteria	Parcubacteria	Candidatus_Campbellbacteria	Candidatus_Campbellbacteria	0.939	0.0014	1	OTU584
BCR	Bacteria	Pseudomonadota	Gammaproteobacteria	Diplorickettsiales	Diplorickettsiaceae	0.938	0.0014	1	OTU852
BCR	Bacteria	Pseudomonadota	Gammaproteobacteria	Burkholderiales	Hydrogenophilaceae	0.937	0.0014	2	OTU830
BCR	Bacteria	Bacteroidota	Bacteroidia	Flavobacteriales	Crocinitomicaceae	0.932	0.0014	1	OTU227
BCR	Bacteria	Actinomycetota	Thermoleophilia	Solirubrobacterales	Actinomycetota_67-14	0.919	0.0014	3	OTU158
BCR	Bacteria	Campilobacterota	Campylobacteria	Campylobacterales	Arcobacteraceae	0.919	0.0014	1	OTU320
BCR	Bacteria	Chloroflexota	KD4-96	KD4-96	Chloroflexota_KD4-96	0.917	0.0014	4	OTU359
BCR	Bacteria	Bacillota	Bacilli	Paenibacillales	Paenibacillaceae	0.917	0.0014	2	OTU426
BCR	Bacteria	Actinomycetota	Thermoleophilia	Gaiellales	Gaiellaceae	0.914	0.0014	1	OTU142
BCR	Bacteria	Pseudomonadota	Gammaproteobacteria	Legionellales	Legionellaceae	0.913	0.0014	1	OTU867
BCR	Bacteria	Desulfobacterota	Desulfuromonadia	Sva1033	Desulfobacterota_Sva1033	0.912	0.0014	1	OTU391
BCR	Bacteria	Pseudomonadota	Gammaproteobacteria	Xanthomonadales	Xanthomonadaceae	0.911	0.0014	2	OTU919
BCR	Bacteria	Bacillota	Clostridia	Caldicoprobacterales	Caldicoprobacteraceae	0.905	0.0014	1	OTU436
BCR	Bacteria	Deinococcota	Deinococci	Deinococcales	Trueperaceae	0.904	0.0014	2	OTU375
BCR	Bacteria	Verrucomicrobiota	Verrucomicrobiae	Chthoniobacterales	Chthoniobacteraceae	0.894	0.0014	1	OTU951
BCR	Bacteria	Babelota	Babeliae	Babeliales	Vermiphilaceae	0.892	0.0014	1	OTU382
BCR	Bacteria	Actinomycetota	Acidimicrobiia	IMCC26256	Actinomycetota_IMCC26256	0.89	0.0014	1	OTU60
BCR	Bacteria	Planctomycetota	Planctomycetes	Pirellulales	Pirellulaceae	0.89	0.0014	1	OTU728
BCR	Bacteria	Chloroflexota	Gitt-GS-136	Gitt-GS-136	Chloroflexota_Gitt-GS-136	0.889	0.0014	1	OTU350
BCR	Bacteria	Pseudomonadota	Gammaproteobacteria	Burkholderiales	Gallionellaceae	0.886	0.0014	1	OTU828
BCR	Bacteria	Pseudomonadota	Gammaproteobacteria	Oceanospirillales	Pseudohongiellaceae	0.881	0.0014	2	OTU879
BCR	Bacteria	Verrucomicrobiota	Lentisphaeria	SS1-B-02-17	Verrucomicrobiota_SS1-B-02-17	0.879	0.0014	1	OTU944
BCR	Bacteria	Pseudomonadota	Gammaproteobacteria	Thiotrichales	Thiotrichaceae	0.868	0.0014	2	OTU901
BCR	Bacteria	Patescibacteria	Berkelbacteria	Berkelbacteria	Berkelbacteria_Incertae_sedis	0.864	0.0007	4	OTU545
BCR	Bacteria	Patescibacteria	Parcubacteria	Candidatus_Nomurabacteria	Candidatus_Nomurabacteria	0.864	0.0014	2	OTU610
BCR	Bacteria	Actinomycetota	Actinobacteria	Frankiales	Sporichthyaceae	0.864	0.0014	1	OTU91
BCR	Bacteria	Actinomycetota	Actinobacteria	Frankiales	Geodermatophilaceae	0.861	0.0014	1	OTU90

BCR	Bacteria	Pseudomonadota	Gammaproteobacteria	Gammaproteobacteria_Incertae_Sedis	Pseudomonadota_Unknown_Family	0.861	0.0014	2	OTU862
BCR	Bacteria	Patescibacteria	Parcubacteria	Candidatus_Moranbacteria	Candidatus_Moranbacteria	0.852	0.0014	1	OTU598
BCR	Bacteria	Patescibacteria	WWE3	WWE3	Patescibacteria_WWE3	0.847	0.0023	1	OTU698
BCR	Bacteria	Chloroflexota	Chloroflexia	Thermomicrobiales	Chloroflexota_JG30-KF-CM45	0.846	0.0014	4	OTU348
BCR	Bacteria	Patescibacteria	ABY1	Candidatus_Falkowbacteria	Candidatus_Falkowbacteria	0.844	0.0023	1	OTU528
BCR	Bacteria	Pseudomonadota	Alphaproteobacteria	Rhodobacterales	Rhodobacteraceae	0.839	0.0014	3	OTU761
BCR	Bacteria	Bacteroidota	Kapabacteria	Kapabacteriales	Kapabacteriales_Incertae_sedis	0.832	0.012	1	OTU308
BCR	Bacteria	Bacillota	Clostridia	Peptostreptococcales-Tissierellales	Anaerovoracaceae	0.83	0.012	1	OTU463
BCR	Bacteria	Patescibacteria	Saccharimonadia	Saccharimonadales	Patescibacteria_LWQ8	0.828	0.012	4	OTU625
BCR	Bacteria	Campilobacterota	Campylobacteria	Campylobacterales	Sulfurimonadaceae	0.825	0.0014	3	OTU323
BCR	Bacteria	Patescibacteria	Saccharimonadia	Saccharimonadales	Patescibacteria_WWH38	0.823	0.0101	1	OTU695
BCR	Bacteria	Actinomycetota	Acidimicrobiia	Microtrichales	Microtrichaceae	0.821	0.0118	2	OTU73
BCR	Bacteria	Verrucomicrobiota	Chlamydiae	Chlamydiales	Parachlamydiaceae	0.82	0.012	1	OTU934
BCR	Bacteria	Pseudomonadota	Gammaproteobacteria	Burkholderiales	Burkholderiaceae	0.819	0.012	1	OTU817
BCR	Bacteria	Patescibacteria	ABY1	Candidatus_Kerfeldbacteria	Candidatus_Kerfeldbacteria	0.817	0.0118	1	OTU534
BCR	Bacteria	Acidobacteriota	Acidobacteriae	PAUC26f	Acidobacteriota_PAUC26f	0.799	0.0014	1	OTU27
BCR	Bacteria	Bacteroidota	Bacteroidia	Sphingobacteriales	Lentimicrobiaceae	0.798	0.0041	2	OTU290
BCR	Bacteria	Verrucomicrobiota	Verrucomicrobiae	Chthoniobacterales	Terrimicrobiaceae	0.794	0.0014	1	OTU958
BCR	Bacteria	Pseudomonadota	Alphaproteobacteria	Rhizobiales	Devosiaceae	0.792	0.012	1	OTU748
BCR	Bacteria	Bacillota	Bacilli	RF39	Bacillota_RF39	0.788	0.0101	1	OTU427
BCR	Bacteria	Pseudomonadota	Gammaproteobacteria	Cellvibrionales	Porticoccaceae	0.787	0.0014	1	OTU846
BCR	Bacteria	Pseudomonadota	Gammaproteobacteria	Oceanospirillales	Nitrincolaceae	0.785	0.0101	1	OTU875
BCR	Bacteria	Verrucomicrobiota	Verrucomicrobiae	Verrucomicrobiales	Rubritaleaceae	0.767	0.0118	1	OTU981
BCR	Bacteria	Bdellovibrionota	Bdellovibrionia	Bacteriovoracales	Bacteriovoracaceae	0.759	0.0122	1	OTU314
BCR	Bacteria	Verrucomicrobiota	Verrucomicrobiae	Verrucomicrobiales	Verrucomicrobiota_DEV007	0.759	0.0026	2	OTU976
BCR	Bacteria	Patescibacteria	Gracilibacteria	Gracilibacteria	Gracilibacteria_Incertae_sedis	0.754	0.0118	1	OTU559
BCR	Bacteria	Pseudomonadota	Gammaproteobacteria	Burkholderiales	Alcaligenaceae	0.749	0.0101	1	OTU816
BCR	Bacteria	Pseudomonadota	Gammaproteobacteria	Cellvibrionales	Spongiibacteraceae	0.731	0.0014	1	OTU847
BCR	Bacteria	Actinomycetota	Acidimicrobiia	Microtrichales	Iamiaceae	0.72	0.012	1	OTU64
BCR	Bacteria	Spirochaetota	Spirochaetia	Spirochaetales	Spirochaetaceae	0.713	0.0118	1	OTU927
BCR	Bacteria	Pseudomonadota	Gammaproteobacteria	Alteromonadales	Marinobacteraceae	0.705	0.0233	1	OTU805

BCR	Bacteria	Cyanobacteriota	Cyanobacteriia	Phormidesmiales	Nodosilineaceae	0.636	0.039	1	OTU369

Table S3. List of the significant ($p < 0.05$) fungal indicator species of the brines. IV, indicator value.

Brine	Kingdom	Phylum	Class	Order	Family	IV	p value	N OTU	Representative OTU
B2	Fungi	Monoblepharomycota	Hyaloraphidiomycetes	Hyaloraphidiales	Hyaloraphidiales_fam_Incertae_sedis	0.972	0.0007	1	OTU399
B2	Fungi	Ascomycota	Eurotiomycetes	Eurotiales	Aspergillaceae	0.939	0.0007	1	OTU279
B2	Fungi	Sanchytriomycota	Sanchytriomycetes	Sanchytriales	Sanchytriaceae	0.916	0.0007	1	OTU422
B2	Fungi	Basidiomycota	Microbotryomycetes	Kriegeriales	Camptobasidiaceae	0.886	0.0007	2	OTU145
B2	Fungi	Chytridiomycota	Rhizophydiomycetes	Rhizophydiales	Alphamycetaceae	0.857	0.0007	1	OTU267
B2	Fungi	Ascomycota	Eurotiomycetes	Chaetothyriales	Cyphellophoraceae	0.841	0.0115	1	OTU319
B2	Fungi	Ascomycota	Dothideomycetes	Pleosporales	Coniothyriaceae	0.821	0.0115	1	OTU321
B2	Fungi	Ascomycota	Dothideomycetes	Pleosporales	Didymellaceae	0.8	0.0007	1	OTU246
B2	Fungi	Ascomycota	Dothideomycetes	Dothideales	Dothideales_fam_Incertae_sedis	0.759	0.0092	1	OTU356
B2	Fungi	Ascomycota	Dothideomycetes	Mycosphaerellales	Neodevriesiaceae	0.759	0.0007	1	OTU200
B2	Fungi	Ascomycota	Sordariomycetes	Pleurotheciales	Pleurotheciaceae	0.731	0.0007	1	OTU248
B2	Fungi	Basidiomycota	Tremellomycetes	Cystofilobasidiales	Mrakiaceae	0.725	0.0081	1	OTU155
B2	Fungi	Chytridiomycota	Chytridiomycota_cls_Incertae_sedis	Chytridiomycota_ord_Incertae_sedis	Chytridiomycota_fam_Incertae_sedis	0.705	0.0092	1	OTU34
B2	Fungi	Monoblepharomycota	Monoblepharidomycetes	Monoblepharidales	Monoblepharidaceae	0.698	0.0122	1	OTU273
B2	Fungi	Mucoromycota	Mucoromycetes	Mucorales	Mucoraceae	0.664	0.0092	1	OTU353
B2	Fungi	Ascomycota	Eurotiomycetes	Chaetothyriales	Pyrenotrichaceae	0.654	0.0092	1	OTU111
B2	Fungi	Basidiomycota	Agaricomycetes	Agaricales	Inocybaceae	0.604	0.0115	1	OTU401
B2	Fungi	Ascomycota	Dothideomycetes	Capnodiales	Capnodiales_fam_Incertae_sedis	0.556	0.0027	1	OTU358
BCM	Fungi	Basidiomycota	Tremellomycetes	Cystofilobasidiales	Mrakiaceae	0.923	0.0008	2	OTU53
BCM	Fungi	Ascomycota	Saccharomycetes	Saccharomycetales	Saccharomycetaceae	0.835	0.0008	1	OTU66
BCM	Fungi	Basidiomycota	Cystobasidiomycetes	Cystobasidiales	Cystobasidiaceae	0.826	0.0115	1	OTU298
BCR	Fungi	Ascomycota	Sordariomycetes	Hypocreales	Stachybotryaceae	0.946	0.0008	3	OTU195
BCR	Fungi	Ascomycota	Sordariomycetes	Sordariales	Chaetomiaceae	0.927	0.0008	1	OTU185
BCR	Fungi	Ascomycota	Sordariomycetes	Hypocreales	Nectriaceae	0.923	0.0008	3	OTU340
BCR	Fungi	Ascomycota	Sordariomycetes	Microascales	Microascaceae	0.905	0.0008	1	OTU152
BCR	Fungi	Ascomycota	Pezizomycetes	Pezizales	Pyronemataceae	0.905	0.0008	1	OTU70
BCR	Fungi	Basidiomycota	Tremellomycetes	Tremellales	Bulleribasidiaceae	0.879	0.0008	1	OTU123

BCR	Fungi	Ascomycota	Dothideomycetes	Pleosporales	Sporormiaceae	0.874	0.0008	2	OTU381
BCR	Fungi	Ascomycota	Saccharomycetes	Saccharomycetales	Saccharomycetales_fam_Incertae_sedis	0.872	0.0014	1	OTU397
BCR	Fungi	Ascomycota	Dothideomycetes	Pleosporales	Pleosporaceae	0.863	0.0008	3	OTU129
BCR	Fungi	Chytridiomycota	Spizellomycetes	Spizellomycetales	Powellomycetaceae	0.835	0.0008	2	OTU314
BCR	Fungi	Ascomycota	Pezizomycetes	Pezizales	Pezizaceae	0.831	0.0008	2	OTU213
BCR	Fungi	Ascomycota	Dothideomycetes	Pleosporales	Pleosporales_fam_Incertae_sedis	0.831	0.0008	1	OTU357
BCR	Fungi	Basidiomycota	Agaricostilbomycetes	Agaricostilbales	Chionosphaeraceae	0.794	0.0008	1	OTU362
BCR	Fungi	Basidiomycota	Exobasidiomycetes	Exobasidiales	Brachybasidiaceae	0.731	0.0014	1	OTU120
BCR	Fungi	Ascomycota	Sordariomycetes	Hypocreales	Bionectriaceae	0.724	0.0122	1	OTU122
BCR	Fungi	Mucoromycota	Mucoromycetes	Mucorales	Rhizopodaceae	0.629	0.0259	1	OTU285
BCR	Fungi	Ascomycota	Laboulbeniomycetes	Pyxidiophorales	Pyxidiophoraceae	0.626	0.0251	1	OTU214
BCR	Fungi	Rozellomycota	Rozellomycota_cls_Incertae_sedis	Rozellomycota_ord_Incertae_sedis	Rozellomycota_fam_Incertae_sedis	0.568	0.0077	1	OTU406
BCR	Fungi	Mucoromycota	Mucoromycetes	Mucorales	Mucoraceae	0.528	0.0165	1	OTU360

Table S4. Count of species and ratio of generalist to specialist bacterial taxa in the brine comparisons.

**BCM-BCR**	**Species**	**Proportion**
Generalist	70	0.118
Specialist_BCM	17	0.029
Specialist_BCR	107	0.18
Too_rare	400	0.673
**B2-BCM**		
Generalist	11	0.029
Specialist_B2	44	0.115
Specialist_BCM	43	0.112
Too_rare	286	0.745
**B2-BCR**		
Generalist	20	0.03
Specialist_B2	39	0.058
Specialist_BCR	137	0.204
Too_rare	477	0.709

Table S5. Count of species and ratio of generalist to specialist fungal taxa in the brine comparisons.

**BCM-BCR**	**Species**	**Proportion**
Generalist	18	0.042
Specialist_BCM	4	0.009
Specialist_BCR	5	0.012
Too_rare	402	0.937
**B2-BCR**		
Generalist	4	0.009
Specialist_B2	23	0.054
Specialist_BCM	8	0.019
Too_rare	394	0.918
**B2-BCR**		
Generalist	4	0.009
Specialist_B2	26	0.061
Specialist_BCR	19	0.044
Too_rare	380	0.886

Table S6. List of the significant ($p < 0.05$) correlations among the most abundant bacterial genera (relative abundance > 1%) and abiotic parameters visualized in Figure S5. Ag: silver; As: arsenic; B: boron; Ca: calcium; Cd: cadmium; Cl: chlorine; Co: cobalt; Cr: chromium; Ctot: total carbon; Cu: copper; Fe: iron; K: potassium; Mg: magnesium; Mn: manganese; Na: Sodium; Ni: nickel; Ntot: total nitrogen; P: phosphorus; Pb: lead; Se: selenium; SO4: sulfate; U: uranium; V: vanadium.

**Abiotic parameters**	**Bacterial genera**	**corr**	**p**
Ag	Candidatus_Campbellbacteria	0.86083416	3.79E-05
Ag	*Flavimarina*	0.882201308	1.35E-05
Ag	*Gillisia*	0.780071114	0.000602595
Ag	*Luteolibacter*	0.877602196	1.71E-05
Ag	*Marinobacter*	0.84468066	7.42E-05
Ag	*Psychrobacter*	0.876618877	1.80E-05
Ag	*Psychroflexus*	0.866516764	2.93E-05
As	Bacteroidetes_VC2.1	0.884786646	1.18E-05
As	Candidatus_Patescibacteria_TM7a	0.956110245	2.65E-08
As	*Lysobacter*	0.987537694	7.98E-12
As	*Sulfurovum*	0.980558387	1.41E-10
B	Candidatus_Campbellbacteria	0.772626114	0.000733765
B	*Flavimarina*	0.799909008	0.000343202
B	*Gillisia*	0.851571493	5.62E-05
B	*Luteolibacter*	0.787388833	0.000492921
B	*Marinobacter*	0.82744412	0.000140725
B	*Psychrobacter*	0.819049836	0.000187552
B	*Psychroflexus*	0.805997228	0.000285203
Ca	Candidatus_Campbellbacteria	0.891515046	8.10E-06
Ca	*Flavimarina*	0.921894275	1.03E-06
Ca	*Gillisia*	0.965488579	5.68E-09
Ca	*Luteolibacter*	0.910377856	2.45E-06
Ca	*Marinobacter*	0.908268031	2.84E-06
Ca	*Psychrobacter*	0.925888259	7.41E-07
Ca	*Psychroflexus*	0.93028691	5.03E-07
Cd	Candidatus_Campbellbacteria	0.877772699	1.70E-05
Cd	*Flavimarina*	0.893795127	7.09E-06
Cd	*Gillisia*	0.82002409	0.000181538
Cd	*Luteolibacter*	0.911661182	2.24E-06
Cd	*Marinobacter*	0.814999676	0.000214335
Cd	*Psychrobacter*	0.877294042	1.74E-05
Cd	*Psychroflexus*	0.877464145	1.73E-05
Cl	Candidatus_Campbellbacteria	0.712361791	0.002883161
Cl	*Flavimarina*	0.757830501	0.001063289
Cl	*Gillisia*	0.633303782	0.011260818
Cl	*Luteolibacter*	0.74786193	0.00134617
Cl	*Marinobacter*	0.723827396	0.002282325
Cl	*Psychrobacter*	0.761265806	0.000977791
Cl	*Psychroflexus*	0.746424352	0.001391546
Co	*Luteimonas*	0.528668139	0.042752288
Cr	Bacteroidetes_VC2.1	0.858867678	4.13E-05

Cr	Candidatus_Patescibacteria_TM7a	0.962857528	9.09E-09
Cr	*Lysobacter*	0.988679569	4.28E-12
Cr	*Sulfurovum*	0.967372749	3.96E-09
Ctot	Acidobacteriota_Subgroup_7	0.569072406	0.026827545
Ctot	Candidatus_Patescibacteria_TM7a	0.665804678	0.006737945
Ctot	*Lysobacter*	0.56560129	0.027984366
Cu	*Bacteroidetes_VC2.1*	0.882187024	1.35E-05
Cu	Candidatus_Patescibacteria_TM7a	0.976365003	4.98E-10
Cu	*Lysobacter*	0.981451486	1.04E-10
Cu	*Sulfurovum*	0.960617954	1.32E-08
Fe	*Bacteroidetes_VC2.1*	0.866904705	2.88E-05
Fe	Candidatus_Patescibacteria_TM7a	0.983246049	5.40E-11
Fe	*Lysobacter*	0.966747013	4.47E-09
Fe	*Sulfurovum*	0.947173978	8.64E-08
K	Candidatus_Campbellbacteria	0.910133453	2.49E-06
K	*Flavimarina*	0.976601647	4.66E-10
K	*Gillisia*	0.936625383	2.75E-07
K	*Luteolibacter*	0.95097151	5.37E-08
K	*Marinobacter*	0.960296522	1.39E-08
K	*Psychrobacter*	0.972978492	1.18E-09
K	*Psychroflexus*	0.976567695	4.71E-10
Mg	Candidatus_Campbellbacteria	0.894035908	7.00E-06
Mg	*Flavimarina*	0.905500301	3.42E-06
Mg	*Gillisia*	0.873972808	2.05E-05
Mg	*Luteolibacter*	0.906695601	3.16E-06
Mg	*Marinobacter*	0.901380838	4.47E-06
Mg	*Psychrobacter*	0.918236159	1.38E-06
Mg	*Psychroflexus*	0.894503126	6.81E-06
Mn	Candidatus_Campbellbacteria	0.852680319	5.37E-05
Mn	*Flavimarina*	0.882382856	1.34E-05
Mn	*Gillisia*	0.948614626	7.25E-08
Mn	*Luteolibacter*	0.865482505	3.07E-05
Mn	*Marinobacter*	0.873125207	2.14E-05
Mn	*Psychrobacter*	0.881034184	1.44E-05
Mn	*Psychroflexus*	0.895090844	6.57E-06
Na	Candidatus_Campbellbacteria	0.769325037	0.000798899
Na	*Flavimarina*	0.831844151	0.00012032
Na	*Gillisia*	0.761213727	0.000979044
Na	*Luteolibacter*	0.820261172	0.000180099
Na	*Marinobacter*	0.825983454	0.000148096
Na	*Psychrobacter*	0.855250438	4.82E-05
Na	*Psychroflexus*	0.827329022	0.000141295
Ni	Candidatus_Campbellbacteria	0.848743174	6.31E-05
Ni	*Flavimarina*	0.904031698	3.77E-06
Ni	*Gillisia*	0.819451743	0.000185052
Ni	*Luteolibacter*	0.860316056	3.87E-05
Ni	*Marinobacter*	0.922608668	9.74E-07

Ni	*Psychrobacter*	0.911352028	2.29E-06
Ni	*Psychroflexus*	0.901885503	4.33E-06
Ntot	Candidatus_Campbellbacteria	0.953281688	3.95E-08
Ntot	*Flavimarina*	0.98770017	7.33E-12
Ntot	*Gillisia*	0.961365785	1.17E-08
Ntot	*Luteolibacter*	0.956166463	2.63E-08
Ntot	*Marinobacter*	0.99152969	6.55E-13
Ntot	*Psychrobacter*	0.989818606	2.16E-12
Ntot	*Psychroflexus*	0.990174832	1.71E-12
P	Bacteroidetes_VC2.1	0.86525532	3.10E-05
P	Candidatus_Patescibacteria_TM7a	0.949989443	6.09E-08
P	*Lysobacter*	0.994723181	3.04E-14
P	*Sulfurovum*	0.974087851	9.00E-10
Pb	Bacteroidetes_VC2.1	0.898055063	5.50E-06
Pb	Candidatus_Patescibacteria_TM7a	0.958895693	1.74E-08
Pb	*Lysobacter*	0.983608301	4.69E-11
Pb	*Sulfurovum*	0.970895292	1.90E-09
Se	Bacteroidetes_VC2.1	0.893600234	7.18E-06
Se	Candidatus_Patescibacteria_TM7a	0.918869171	1.31E-06
Se	*Lysobacter*	0.967228561	4.07E-09
Se	*Sulfurovum*	0.956830045	2.38E-08
SO4	Acidobacteriota_Subgroup_7	0.63044439	0.011750159
SO4	*Luteimonas*	0.874093266	2.04E-05
SO4	*Lutibacter*	0.864146936	3.26E-05
U	Candidatus_Campbellbacteria	0.949648999	6.36E-08
U	*Flavimarina*	0.927984681	6.18E-07
U	*Gillisia*	0.965979241	5.18E-09
U	*Luteolibacter*	0.945319356	1.08E-07
U	*Marinobacter*	0.868687636	2.65E-05
U	*Psychrobacter*	0.916206631	1.61E-06
U	*Psychroflexus*	0.927684611	6.34E-07
V	Candidatus_Campbellbacteria	0.886155289	1.09E-05
V	*Flavimarina*	0.945240775	1.09E-07
V	*Gillisia*	0.954543715	3.31E-08
V	*Luteolibacter*	0.941827444	1.60E-07
V	*Marinobacter*	0.927004738	6.73E-07
V	*Psychrobacter*	0.950950592	5.38E-08
V	*Psychroflexus*	0.94193965	1.58E-07
Ag	Acidobacteriota_Subgroup_7	-0.910264616	2.47E-06
Ag	*Luteimonas*	-0.743085024	0.001501722
Ag	*Lutibacter*	-0.748192103	0.001335919
As	*Flavimarina*	-0.515461282	0.049229572
As	*Gillisia*	-0.653185113	0.008281394
As	*Marinobacter*	-0.516923392	0.048478989
As	*Psychroflexus*	-0.530931007	0.041709304
B	Acidobacteriota_Subgroup_7	-0.681386172	0.005154924
B	Bacteroidetes_VC2.1	-0.610381029	0.015665142

B	Candidatus_Patescibacteria_TM7a	-0.782188587	0.000569001
B	*Lysobacter*	-0.678960907	0.005379736
B	*Sulfurovum*	-0.659552733	0.007471367
Ba	*Luteimonas*	-0.79911917	0.000351386
Ba	*Lutibacter*	-0.786780604	0.000501366
Ca	Acidobacteriota_Subgroup_7	-0.791596112	0.000437626
Ca	Bacteroidetes_VC2.1	-0.622610654	0.013175747
Ca	Candidatus_Patescibacteria_TM7a	-0.820079233	0.000181203
Ca	*Lysobacter*	-0.716568335	0.002649608
Ca	*Sulfurovum*	-0.712939793	0.00285013
Cd	Acidobacteriota_Subgroup_7	-0.89302814	7.42E-06
Cd	*Luteimonas*	-0.583045865	0.022533153
Cd	*Lutibacter*	-0.63838057	0.010431212
Cl	Acidobacteriota_Subgroup_7	-0.833453258	0.000113495
Cl	*Luteimonas*	-0.802479624	0.00031764
Cl	*Lutibacter*	-0.796397299	0.000380817
Co	Bacteroidetes_VC2.1	-0.838528947	9.40E-05
Co	Candidatus_Patescibacteria_TM7a	-0.859681877	3.98E-05
Co	*Lysobacter*	-0.925964247	7.36E-07
Co	*Sulfurovum*	-0.927895522	6.23E-07
Cr	*Gillisia*	-0.642490699	0.009795148
Cr	*Psychroflexus*	-0.518393407	0.047732885
Ctot	Candidatus_Campbellbacteria	-0.627583522	0.012256138
Ctot	*Flavimarina*	-0.69855644	0.003767546
Ctot	*Gillisia*	-0.717814147	0.002583426
Ctot	*Luteolibacter*	-0.663369105	0.007016594
Ctot	*Marinobacter*	-0.728377987	0.002073725
Ctot	*Psychrobacter*	-0.704888703	0.003338455
Ctot	*Psychroflexus*	-0.703789355	0.003410009
Cu	Candidatus_Campbellbacteria	-0.546805889	0.0349155
Cu	*Flavimarina*	-0.553514485	0.032308586
Cu	*Gillisia*	-0.68759007	0.004613556
Cu	*Luteolibacter*	-0.542336176	0.036737802
Cu	*Marinobacter*	-0.554659839	0.031878556
Cu	*Psychrobacter*	-0.5438914	0.03609586
Cu	*Psychroflexus*	-0.569271233	0.026762396
EC	*Luteimonas*	-0.521416338	0.046225263
EC	*Lutibacter*	-0.555439968	0.031588127
Fe	Candidatus_Campbellbacteria	-0.62602507	0.012538796
Fe	*Flavimarina*	-0.635374294	0.010916494
Fe	*Gillisia*	-0.756745175	0.001091523
Fe	*Luteolibacter*	-0.623642352	0.012980701
Fe	*Marinobacter*	-0.6361971	0.010781962
Fe	*Psychrobacter*	-0.628647503	0.012066023
Fe	*Psychroflexus*	-0.650131258	0.008693517
K	Acidobacteriota_Subgroup_7	-0.924946182	8.02E-07
K	Candidatus_Patescibacteria_TM7a	-0.591321368	0.020249907

K	*Lutibacter*	-0.526684763	0.043682251
Mg	Acidobacteriota_Subgroup_7	-0.889512944	9.08E-06
Mg	Candidatus_Patescibacteria_TM7a	-0.538058575	0.038547456
Mg	*Luteimonas*	-0.542274922	0.036763259
Mg	*Lutibacter*	-0.55881357	0.030355048
Mn	Acidobacteriota_Subgroup_7	-0.705490664	0.003299785
Mn	Bacteroidetes_VC2.1	-0.735766462	0.001767844
Mn	Candidatus_Patescibacteria_TM7a	-0.912000313	2.19E-06
Mn	*Lysobacter*	-0.835114895	0.000106786
Mn	*Sulfurovum*	-0.823221665	0.000162901
Na	Acidobacteriota_Subgroup_7	-0.853773149	5.13E-05
Na	*Luteimonas*	-0.641444232	0.009954139
Na	*Lutibacter*	-0.668570327	0.006432175
Ni	Acidobacteriota_Subgroup_7	-0.93403516	3.55E-07
Ni	*Luteimonas*	-0.698670352	0.003759458
Ni	*Lutibacter*	-0.719473915	0.002497315
Ntot	Acidobacteriota_Subgroup_7	-0.93674359	2.72E-07
Ntot	Candidatus_Patescibacteria_TM7a	-0.633293724	0.011262511
Ntot	*Lutibacter*	-0.537716197	0.038695119
P	*Gillisia*	-0.610029199	0.015741763
Pb	*Gillisia*	-0.638845296	0.01035772
Rb	Acidobacteriota_Subgroup_7	-0.696640871	0.003905641
Rb	*Luteimonas*	-0.89904622	5.17E-06
Rb	*Lutibacter*	-0.866329493	2.95E-05
Se	*Gillisia*	-0.538080134	0.038538171
SO4	*Lysobacter*	-0.535736812	0.039557081
U	Acidobacteriota_Subgroup_7	-0.761420703	0.000974071
U	Bacteroidetes_VC2.1	-0.636530308	0.01072785
U	Candidatus_Patescibacteria_TM7a	-0.81893887	0.000188248
U	*Lysobacter*	-0.723306886	0.002307219
U	*Sulfurovum*	-0.714735389	0.002749439
V	Acidobacteriota_Subgroup_7	-0.826560488	0.000145147
V	Bacteroidetes_VC2.1	-0.565500914	0.02801837
V	Candidatus_Patescibacteria_TM7a	-0.752125986	0.001218553
V	*Lysobacter*	-0.640043581	0.010170087
V	*Sulfurovum*	-0.636227732	0.010776978

Table S7. List of the significant ($p < 0.05$) correlations among the most abundant fungal genera (relative abundance > 1%) and abiotic parameters visualized in Figure S6. Ag: silver; As: arsenic; B: boron; Ca: calcium; Cd: cadmium; Cl: chlorine; Co: cobalt; Cr: chromium; Ctot: total carbon; Cu: copper; EC: electrical conductivity; Fe: iron; K: potassium; Mg: magnesium; Mn: manganese; Na: Sodium; Ni: nickel; Ntot: total nitrogen; P: phosphorus; Pb: lead; Rb: rubidium; Se: selenium; SO4: sulfate; U: uranium; V: vanadium.

**Abiotic parameters**	**Fungal genera**	**corr**	**p**
Ag	*Betamyces*	0.847656487	6.59E-05
Ag	*Glaciozyma*	0.767209306	0.000843056
Ag	*Penicillium*	0.839186685	9.17E-05
Ag	*Phoma*	0.63634707	0.010757581
Ag	*Saccharomyces*	0.554882185	0.031795577
Ag	*Sanchytrium*	0.806475728	0.000281008
As	*Mrakia*	0.959539646	1.57E-08
B	*Betamyces*	0.611649622	0.015391256
B	*Glaciozyma*	0.580647507	0.023230162
B	*Inocybe*	0.538684432	0.038278618
B	*Penicillium*	0.670407674	0.00623516
B	*Phoma*	0.836523356	0.000101357
B	*Sanchytrium*	0.705874124	0.003275339
Ba	*Saccharomyces*	0.633011404	0.011310113
Ca	*Betamyces*	0.7922833	0.000429096
Ca	*Glaciozyma*	0.768440901	0.000817117
Ca	*Inocybe*	0.643327062	0.009669509
Ca	*Penicillium*	0.83382749	0.000111955
Ca	*Phoma*	0.786373142	0.000507089
Ca	*Sanchytrium*	0.853934218	5.10E-05
Cd	*Betamyces*	0.919484196	1.25E-06
Cd	*Glaciozyma*	0.852520625	5.40E-05
Cd	*Inocybe*	0.600978882	0.017813988
Cd	*Penicillium*	0.866009871	3.00E-05
Cd	*Phoma*	0.568797362	0.026917865
Cd	*Sanchytrium*	0.818819293	0.000188999
Cl	*Betamyces*	0.694478055	0.004066367
Cl	*Glaciozyma*	0.664260437	0.006913587
Cl	*Inocybe*	0.537207586	0.038915253
Cl	*Penicillium*	0.728889869	0.002051252
Cl	*Phoma*	0.56168558	0.029334195
Cl	*Saccharomyces*	0.572497777	0.025721836
Cl	*Sanchytrium*	0.694995782	0.004027423
Co	*Vishniacozyma*	0.560267505	0.029834957
Cr	*Mrakia*	0.949571095	6.43E-08
Ctot	*Mrakia*	0.548522492	0.03423395
Cu	*Mrakia*	0.967943225	3.54E-09
EC	*Saccharomyces*	0.593453023	0.019691538
Fe	*Mrakia*	0.944085775	1.24E-07
K	*Betamyces*	0.877080198	1.76E-05
K	*Glaciozyma*	0.790649887	0.000449598

K	*Inocybe*	0.672015508	0.006066688
K	*Penicillium*	0.966465831	4.72E-09
K	*Phoma*	0.719868155	0.002477203
K	*Saccharomyces*	0.53169044	0.041363545
K	*Sanchytrium*	0.86878963	2.63E-05
Mg	*Betamyces*	0.7762241	0.000667752
Mg	*Glaciozyma*	0.722895212	0.002327062
Mg	*Inocybe*	0.543247857	0.036360466
Mg	*Penicillium*	0.772961746	0.000727391
Mg	*Phoma*	0.856621346	4.55E-05
Mg	*Sanchytrium*	0.814769582	0.000215946
Mn	*Betamyces*	0.762743616	0.000942767
Mn	*Glaciozyma*	0.731670735	0.001932556
Mn	*Inocybe*	0.587870461	0.021179422
Mn	*Penicillium*	0.813456474	0.00022533
Mn	*Phoma*	0.730131701	0.001997544
Mn	*Sanchytrium*	0.832222064	0.000118688
Na	*Betamyces*	0.655931888	0.007923942
Na	*Glaciozyma*	0.664391075	0.00689859
Na	*Inocybe*	0.698113815	0.003799104
Na	*Penicillium*	0.736742704	0.001730302
Na	*Phoma*	0.800207152	0.000340154
Na	*Saccharomyces*	0.545347932	0.035502297
Na	*Sanchytrium*	0.736563251	0.001737154
Ni	*Betamyces*	0.758599977	0.001043632
Ni	*Glaciozyma*	0.676607444	0.005605235
Ni	*Penicillium*	0.86432117	3.24E-05
Ni	*Phoma*	0.749195498	0.001305152
Ni	*Saccharomyces*	0.726512183	0.002157318
Ni	*Sanchytrium*	0.817192654	0.00019947
Ntot	*Betamyces*	0.853809991	5.12E-05
Ntot	*Glaciozyma*	0.784883981	0.000528458
Ntot	*Inocybe*	0.551301288	0.033151877
Ntot	*Penicillium*	0.918585646	1.34E-06
Ntot	*Phoma*	0.815145578	0.000213318
Ntot	*Saccharomyces*	0.590894679	0.020363115
Ntot	*Sanchytrium*	0.919285306	1.27E-06
P	*Mrakia*	0.97630235	5.06E-10
Pb	*Mrakia*	0.971990036	1.48E-09
Rb	*Saccharomyces*	0.572631637	0.025679339
Se	*Mrakia*	0.965458926	5.71E-09
SO4	*Fusarium*	0.854272298	5.02E-05
SO4	*Myrothecium*	0.906841762	3.13E-06
SO4	*Vishniacozyma*	0.890800911	8.44E-06
U	*Betamyces*	0.899228737	5.11E-06
U	*Glaciozyma*	0.880144456	1.50E-05
U	*Inocybe*	0.54530874	0.035518172

U	*Penicillium*	0.820004336	0.000181659
U	*Phoma*	0.710764572	0.002976033
U	*Sanchytrium*	0.914720032	1.79E-06
V	*Betamyces*	0.824069831	0.00015823
V	*Glaciozyma*	0.758415012	0.00104833
V	*Inocybe*	0.74535977	0.001425937
V	*Penicillium*	0.885598795	1.13E-05
V	*Phoma*	0.795973321	0.000385577
V	*Sanchytrium*	0.807744712	0.000270128
Ag	*Fusarium*	-0.92306085	9.38E-07
Ag	*Myrothecium*	-0.895782665	6.31E-06
Ag	*Vishniacozyma*	-0.679745001	0.005306222
B	*Mrakia*	-0.649260233	0.008813957
Ba	*Fusarium*	-0.712509077	0.002874715
Ba	*Myrothecium*	-0.759821469	0.001013032
Ba	*Vishniacozyma*	-0.806068513	0.000284575
Ca	*Mrakia*	-0.654728931	0.008078968
Cd	*Fusarium*	-0.821087395	0.000175156
Cd	*Myrothecium*	-0.760783869	0.000989438
Cd	*Vishniacozyma*	-0.541217618	0.037204751
Cl	*Fusarium*	-0.935362612	3.12E-07
Cl	*Myrothecium*	-0.926349054	7.12E-07
Cl	*Vishniacozyma*	-0.750702566	0.001260017
Co	*Mrakia*	-0.897110942	5.82E-06
Ctot	*Betamyces*	-0.560807415	0.029643546
Ctot	*Penicillium*	-0.694396322	0.004072543
Ctot	*Phoma*	-0.587278914	0.021341975
Ctot	*Sanchytrium*	-0.58413847	0.022220959
Cu	*Penicillium*	-0.516508065	0.048691336
Cu	*Sanchytrium*	-0.514708443	0.049619366
EC	*Fusarium*	-0.634760759	0.011017657
EC	*Myrothecium*	-0.589987291	0.020605468
Fe	*Betamyces*	-0.554569206	0.031912428
Fe	*Penicillium*	-0.583257027	0.022472558
Fe	*Phoma*	-0.530415617	0.041945174
Fe	*Sanchytrium*	-0.593846033	0.019589893
K	*Fusarium*	-0.758972176	0.00103423
K	*Myrothecium*	-0.695058986	0.004022689
Mg	*Fusarium*	-0.72189493	0.002375846
Mg	*Myrothecium*	-0.674011771	0.005862535
Mn	*Mrakia*	-0.792898389	0.000421576
Na	*Fusarium*	-0.883287836	1.28E-05
Na	*Myrothecium*	-0.836372053	0.000101929
Na	*Vishniacozyma*	-0.569739874	0.026609314
Ni	*Fusarium*	-0.89361638	7.17E-06
Ni	*Myrothecium*	-0.861575105	3.66E-05
Ni	*Vishniacozyma*	-0.639869728	0.010197144

Ntot	*Fusarium*	-0.758685054	0.001041477
Ntot	*Myrothecium*	-0.697464842	0.003845755
Rb	*Fusarium*	-0.891873346	7.93E-06
Rb	*Myrothecium*	-0.923937437	8.73E-07
Rb	*Vishniacozyma*	-0.858085272	4.27E-05
Se	*Vishniacozyma*	-0.561869092	0.029269858
SO4	*Mrakia*	-0.547263387	0.034732872
SO4	*Saccharomyces*	-0.562241104	0.029139763
U	*Mrakia*	-0.679783045	0.005302676
V	*Fusarium*	-0.59324736	0.01974489
V	*Mrakia*	-0.597287566	0.018716873
V	*Myrothecium*	-0.514794567	0.049574659
